# Group II Intron Protein Localization and Insertion Sites Are Affected by Polyphosphate

**DOI:** 10.1371/journal.pbio.0060150

**Published:** 2008-06-24

**Authors:** Junhua Zhao, Wei Niu, Jun Yao, Sabine Mohr, Edward M Marcotte, Alan M Lambowitz

**Affiliations:** 1 Institute for Cellular and Molecular Biology, University of Texas at Austin, Austin, Texas, United States of America; 2 Department of Chemistry and Biochemistry, University of Texas at Austin, Austin, Texas, United States of America; 3 Section of Molecular Genetics and Microbiology, School of Biological Sciences, University of Texas at Austin, Austin, Texas, United States of America; Wadsworth Center, New York State Department of Health, United States of America

## Abstract

Mobile group II introns consist of a catalytic intron RNA and an intron-encoded protein with reverse transcriptase activity, which act together in a ribonucleoprotein particle to promote DNA integration during intron mobility. Previously, we found that the Lactococcus lactis Ll.LtrB intron-encoded protein (LtrA) expressed alone or with the intron RNA to form ribonucleoprotein particles localizes to bacterial cellular poles, potentially accounting for the intron's preferential insertion in the *oriC* and *ter* regions of the Escherichia coli chromosome. Here, by using cell microarrays and automated fluorescence microscopy to screen a transposon-insertion library, we identified five E. coli genes (*gppA*, *uhpT*, *wcaK*, *ynbC*, and *zntR*) whose disruption results in both an increased proportion of cells with more diffuse LtrA localization and a more uniform genomic distribution of Ll.LtrB-insertion sites. Surprisingly, we find that a common factor affecting LtrA localization in these and other disruptants is the accumulation of intracellular polyphosphate, which appears to bind LtrA and other basic proteins and delocalize them away from the poles. Our findings show that the intracellular localization of a group II intron-encoded protein is a major determinant of insertion-site preference. More generally, our results suggest that polyphosphate accumulation may provide a means of localizing proteins to different sites of action during cellular stress or entry into stationary phase, with potentially wide physiological consequences.

## Author Summary

Group II introns are bacterial mobile elements thought to be ancestors of introns—genetic material that is discarded from messenger RNA transcripts—and retroelements—genetic elements and viruses that replicate via reverse transcription—in higher organisms. They propagate by forming a complex consisting of the catalytically active intron RNA and an intron-encoded reverse transcriptase (which converts the RNA to DNA, which can then be reinserted in the host genome). The Ll.LtrB group II intron-encoded protein (LtrA) was found previously to localize to bacterial cellular poles, potentially accounting for the preferential insertion of Ll.LtrB in the replication origin (*oriC*) and terminus (*ter*) regions of the Escherichia coli chromosome, which are located near the poles during much of the cell cycle. Here, we identify E. coli genes whose disruption leads both to more diffuse LtrA localization and a more uniform chromosomal distribution of Ll.LtrB-insertion sites, proving that the location of the LtrA protein contributes to insertion-site preference. Surprisingly, we find that LtrA localization in the disruptants is affected by the accumulation of intracellular polyphosphate, which appears to bind basic proteins and delocalize them away from the cellular poles. Thus, polyphosphate, a ubiquitous but enigmatic molecule in prokaryotes and eukaryotes, can localize proteins to different sites of action, with potentially wide physiological consequences.

## Abstract 3

A novel cell microarray method uncovers connections between group II intron mobility, cell stress, and polyphosphate metabolism, including the finding that polyphosphate can influence intracellular protein localization.

## Introduction

Mobile group II introns, found in bacterial and organelle genomes, consist of a catalytic intron RNA and a multifunctional intron-encoded protein (IEP), which interact to promote RNA splicing and intron mobility [1,2]. The IEP binds to the intron in unspliced precursor RNA, promotes its splicing by stabilizing the catalytically active RNA structure, and remains tightly bound to the excised intron lariat RNA in a ribonucleoprotein particle (RNP) that promotes intron mobility. Mobility occurs by a remarkable mechanism in which the intron RNA inserts (reverse splices) directly into a DNA strand and is reverse-transcribed by the IEP, with the primer being either the opposite DNA strand cleaved by the IEP or a nascent strand at a DNA replication fork (reviewed in [1]). By using this reverse splicing mechanism, mobile group II introns insert at high frequency into specific DNA target sites (“retrohoming”) and at low frequency into ectopic sites that resemble the normal homing site (“retrotransposition”) [1]. The latter process has led to the wide dispersal of mobile group II introns among bacterial species and also may have been used to invade eukaryotic nuclear genomes, where mobile group II introns are thought to have evolved into spliceosomal introns and non-long-terminal-repeat retrotransposons [2]. Although their DNA integration mechanism has been elucidated, little is known about how mobile group II introns function in a cellular context or about how their mobility is influenced by other cellular processes.

The Lactococcus lactis Ll.LtrB intron, which has been used as a model system, is highly mobile not only in L. lactis but also in a variety of other bacteria including Escherichia coli, where it has been studied extensively by using the facile genetic and biochemical methods available for that organism [1,3]. The broad host range of the Ll.LtrB intron reflects that RNPs containing only the IEP and intron RNA by themselves carry out the initial reverse splicing and reverse transcription reactions, while the subsequent cDNA integration steps use common host DNA repair functions [4,5].

Ll.LtrB and other mobile group II introns recognize their DNA target sequences by using both the IEP and the base pairing of the intron RNA, with the latter contributing most of the target specificity [6–8]. For Ll.LtrB, these base-pairing interactions involve intron RNA sequences denoted EBS2, EBS1, and δ and complementary DNA target sequences denoted IBS2, IBS1, and δ', extending from position −12 to +2 from the intron-insertion site. (EBS and IBS denote exon- and intron-binding site, respectively.) Because the DNA target site is recognized largely by base pairing of the intron RNA, Ll.LtrB can be retargeted to insert (retrohome) into different chromosomal sites, enabling its development into a gene targeting vector (“targetron”) [6,8,9]. Further, an Ll.LtrB intron with randomized EBS2, EBS1, and δ sequences inserts at sites distributed throughout the E. coli genome, analogous to global transposon mutagenesis. Surprisingly, however, we found that although Ll.LtrB could be retargeted to insert efficiently into any region of the E. coli genome, an Ll.LtrB intron with randomized EBS and δ sequences shows a strong proclivity to insert at sites clustered around the bidirectional replication origin (*oriC*), with 57% of the sites lying within 5% of the chromosome on either side of *oriC* [10]. Coros et al. [11] studying retrotransposition of the wild-type Ll.LtrB intron into ectopic sites in E. coli observed a similar clustering of insertion sites in both the *oriC* and the *ter* regions.

Previously, we hypothesized that the preferential insertion of the Ll.LtrB intron into the *oriC* and *ter* regions of the E. coli chromosome might reflect the intracellular localization of Ll.LtrB RNPs [12]. In E. coli, the *oriC* and *ter* regions are localized near the cellular poles during much of the cell cycle [13,14], and we found by using both LtrA fusions with green fluorescent protein (GFP) and immunofluorescence microscopy that LtrA expressed alone or with Ll.LtrB RNA to form RNPs localizes to the cellular poles in both E. coli and L. lactis [12]. Further analysis in E. coli showed that the bipolar localization of LtrA occurs over a wide range of cellular growth rates and LtrA expression levels, is independent of *oriC* function, and occurs in anucleate cells, suggesting that LtrA simply is not forced to the poles by nucleoid occlusion [12]. We also found that LtrA expression in E. coli interferes with the polar localization of coexpressed Shigella flexneri IcsA protein, suggesting competition for a common localization determinant [12]. Beauregard et al. [15] found that the polar localization of LtrA is maintained in E. coli mutants with defects in nucleoid condensation, chromosome partitioning, and DNA replication, and as expected from its continued pole localization, Ll.LtrB retrotransposition sites remained clustered in the *oriC* and *ter* regions in such mutants.

While the above findings are consistent with the possibility that the bipolar localization of LtrA is responsible for the clustering of Ll.LtrB-insertion sites in the *oriC* and *ter* regions of the E. coli chromosome, to prove this connection, it is necessary to obtain mutations that change LtrA's intracellular localization and show that they correspondingly change the chromosomal distribution of Ll.LtrB-insertion sites. However, mutations that affect the polar localization of proteins in E. coli have been difficult to find. Nilsen et al. [16] manually screened ∼7,000 E. coli mutants for altered localization of the S. flexneri IcsA protein. The only mutant identified was in the *mreB* gene, which encodes a bacterial actin homologue required for maintenance of the cell's rod shape, and in this case, an IcsA/GFP fusion protein simply showed multiple foci in a portion of the spherical mutant cells instead of two foci at poles.

Here, we used automated fluorescence microscopy of cell microarrays [17] to screen a transposon-insertion library for mutants with altered LtrA localization. We identified five E. coli genes (*gppA*, *uhpT*, *wcaK*, *ynbC*, and *zntR*) whose disruption leads to both a more diffuse intracellular distribution of LtrA and a more uniform genomic distribution of Ll.LtrB-insertion sites, indicating that group II intron protein localization is a major determinant of insertion-site preference. Surprisingly, we find that the common factor affecting LtrA localization in these disruptants is the accumulation of intracellular polyphosphate poly(P). We confirmed this connection by analyzing *ppx* disruptants, which lack the exopolyphosphatase that degrades poly(P), and found that disruptants that accumulate poly(P) also show delocalization of other pole-localized basic proteins (Neurospora crassa CYT-18 and E. coli XapR). Our findings show that poly(P), which accumulates in response to cell stress or entry into stationary phase, can localize proteins to different sites of action, with potentially wide physiological consequences.

## Results

### Cell Microarray Screen for E. coli Mutants with Altered LtrA Localization

**Figure 1:**
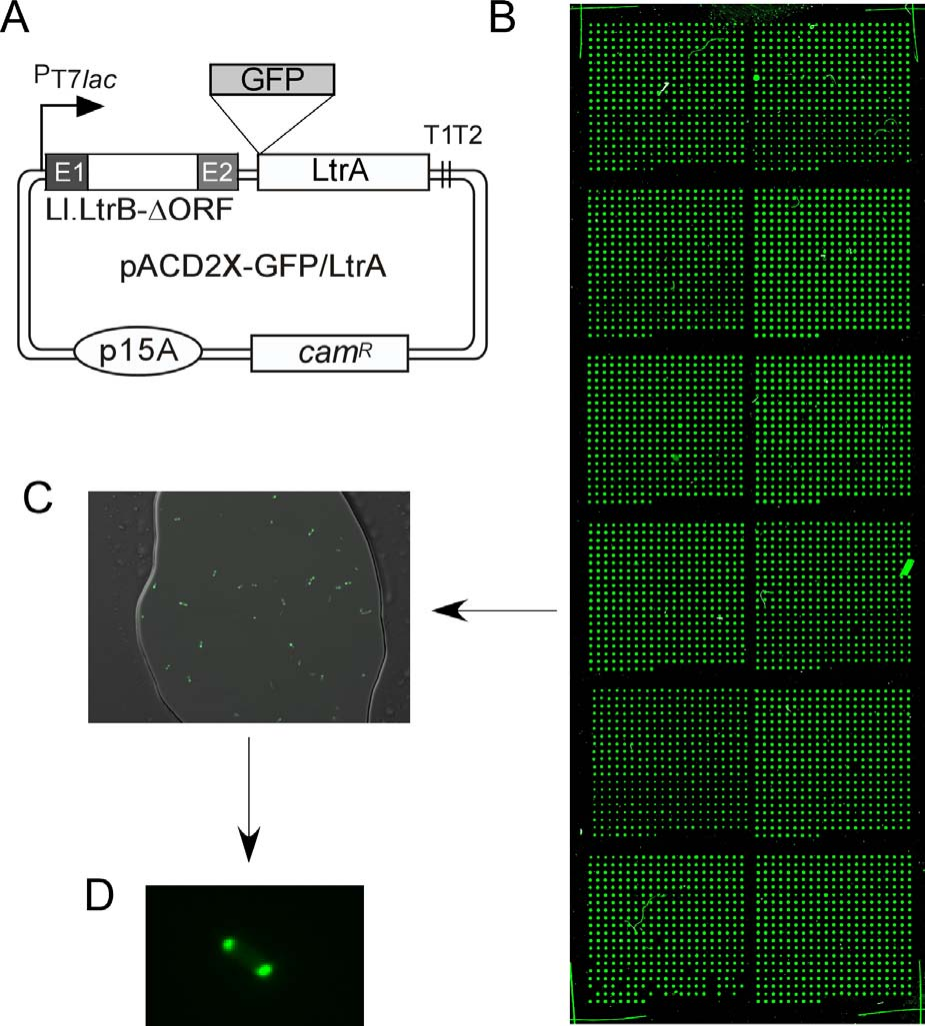
Intron-Expression Plasmid and Cell Microarrays Used to Identify E. coli Disruptants with Altered GFP/LtrA Localization **(**A) pACD2X-GFP/LtrA uses a T7 *lac* promoter to express the Ll.LtrB-ΔORF intron with short flanking exons, followed by a GFP/LtrA fusion protein [12]. E1 and E2 are 5′ and 3′ exons, respectively, and T1 and T2 are *rrnB* transcription terminators. (B) Wide-field light scattering image of a cell microarray. A *mariner* transposon-insertion library of E. coli HMS174(DE3) cells carrying pACD2X-GFP/LtrA was arrayed onto microscope slides like that shown and screened by automated fluorescence microscopy to identify mutants with altered GFP/LtrA localization patterns (see Materials and Methods section). (C) Close-up of a spot from the cell microarray. (D) Higher magnification view of the same spot focusing on an E. coli cell with the wild-type bipolar GFP/LtrA localization pattern.

To screen for E. coli mutants with altered LtrA localization, we constructed a library of *mariner* transposon insertions in E. coli HMS174(DE3), a standard host strain that contains an integrated λDE3 prophage with an isopropyl β-d-1-thiogalactopyranoside (IPTG)-inducible T7 RNA polymerase for Ll.LtrB intron expression (see Materials and Methods section). For screening, the library was transformed with intron-donor plasmid pACD2X-GFP/LtrA, which uses a *T7lac* promoter to express an Ll.LtrB-ΔORF intron with short flanking exons, plus a GFP/LtrA fusion protein downstream of the 3′ exon (E2; Figure 1A). This configuration gives high intron mobility frequencies and permits independent manipulation of the intron RNA and IEP. We showed previously that GFP/LtrA expressed from this plasmid is active in promoting both RNA splicing and intron mobility [12].

The library in 96-well plate format was arrayed onto microscope slides and screened for mutants with altered GFP/LtrA localization by automated fluorescence microscopy (Figure 1B; see Materials and Methods section). The images were stored and then examined individually to characterize GFP/LtrA localization patterns (Figure 1C and 1D). A total of 9,600 disruptants were screened under two different Ll.LtrB induction conditions (overnight with 500 μM IPTG at 30 °C or 100 μM IPTG at 37 °C). Of 277 initial candidates, 36 showed similarly altered GFP/LtrA localization patterns in duplicate arrays, and five that showed the most strongly altered GFP/LtrA localization patterns in liquid culture were studied further.

### Identification of E. coli Genes Affecting LtrA Localization

The *mariner* transposon-insertion sites in the five disruptants were amplified and sequenced via thermal-asymmetric-interlaced (TAIL) PCR and found to be in the *gppA*, *uhpT*, *wcaK*, *ynbC*, and *zntR* genes. *gppA* encodes guanosine pentaphosphatase A, which removes the 5′ phosphate from pppGpp to produce the stringent response regulator, ppGpp (“magic spot”) [18]; *uhpT* encodes a hexose phosphate transport component [19]; *wcaK* is a predicted colanic acid biosynthesis pyruvyl transferase [20]; *ynbC* encodes a 585-amino-acid ORF of unknown function [21]; and *zntR* encodes a zinc-responsive transcriptional regulator [22]. In each disruptant, the transposon-insertion site was confirmed by additional PCRs to amplify and sequence both the 5′- and the 3′-transposon-integration junctions (Figure S1) and by Southern hybridization, which also showed that each strain contains a single transposon insertion (Figure S2). Immunoblots showed that the GFP/LtrA expression levels in the disruptants are similar to or lower than those in the wild-type strain, with the expression level particularly low in the *ynbC* disruptant (Figure S3). These findings indicate that the disruptions affect GFP/LtrA localization over a wide range of protein expression levels. *gppA* and *uhpT* are in single gene transcription units, and *zntR* is the last gene of a two-gene operon, while *ynbC* and *wcaK* are upstream genes in operons whose disruption could exert polar effects on downstream genes [23].

### Intracellular Localization of LtrA in the Disruptants

**Figure 2:**
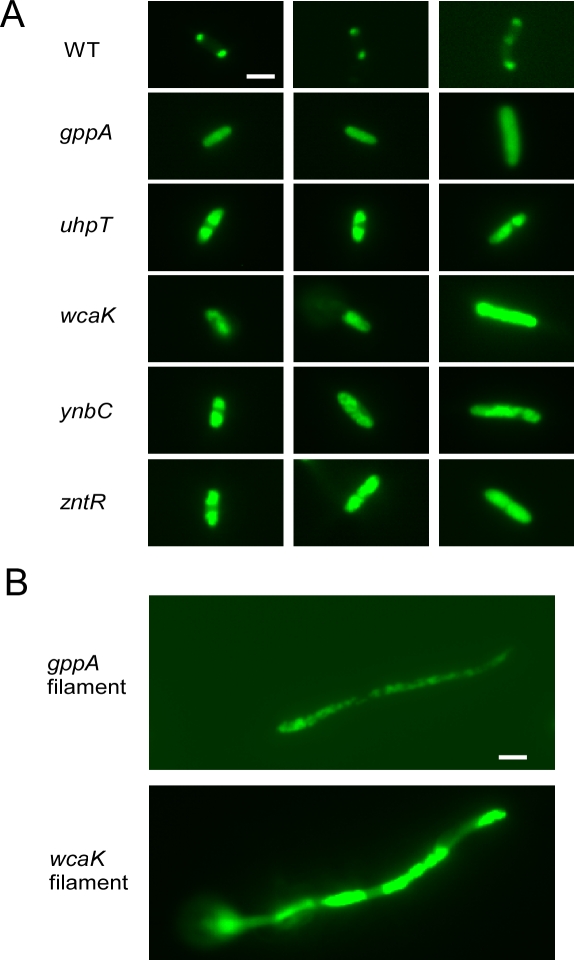
Fluorescence Microscopy of GFP/LtrA in Wild-Type E. coli and Disruptants with Altered GFP/LtrA Localization Patterns Wild-type HMS174(DE3) (WT) and disruptants containing pACD2X-GFP/LtrA were induced with 500 μM IPTG overnight at 30 °C. For each strain, ≥200 cells were examined by fluorescence microscopy, as described in the Materials and Methods section, and the proportions of cells with different LtrA localization patterns are summarized in Table 1, top. The localization patterns shown in the figure represent the most common pattern for each strain. Bar = 2 μm. (A) Wild-type cells showing bipolar GFP/LtrA localization and disruptants showing diffuse GFP/LtrA localization patterns. (B) Filamentous *gppA* and *wcaK* cells showing multiple GFP/LtrA foci, diffuse patches, or a combination of the two.

To characterize their GFP/LtrA localization patterns, the wild-type and disruptant strains were grown in liquid culture, and GFP/LtrA expression was induced overnight with 500 μM IPTG at 30 °C, one of the induction conditions used for screening the transposon-insertion library. GFP/LtrA localization patterns in ≥200 randomly selected cells of each strain were then analyzed by fluorescence microscopy (Figure 2 and Table 1, top). As found previously, in the wild-type strain, a high proportion of the cells (81.2%) showed polar localization of LtrA. Such cells contain one or two small, discrete GFP/LtrA foci at their poles, with elongated cells, presumably those ready for division, typically showing a third focus in the middle. Only 2.8% of the wild-type cells showed diffuse GFP/LtrA localization patterns. In wild type as well as the disruptants, ∼20% of cells did not show GFP/LtrA fluorescence detectable above background.

**Table 1:**
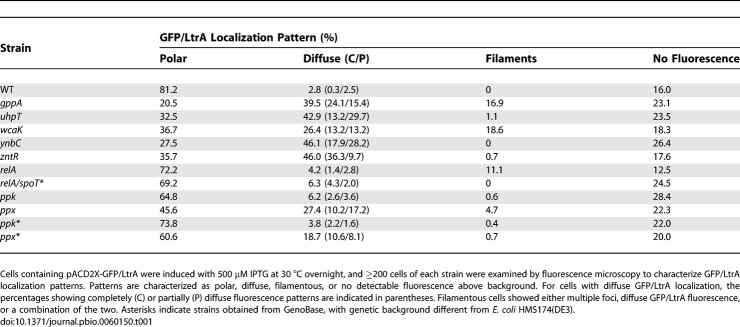
GFP/LtrA Localization in Wild-Type and Mutant Strains

In comparison to the wild-type strain, each of the disruptants showed a substantially increased proportion of cells with more diffuse GFP/LtrA localization patterns (26.4–46.1%), which were classified into two types: completely diffuse (C), with GFP/LtrA uniformly distributed throughout the cell, or partially diffuse (P), with GFP/LtrA spread out from the poles but leaving a clear area in the middle of the cell (percentages of C and P are indicated in parentheses in Table 1). The *gppA* disruptant was the most strongly affected, with only 20.5% of the cells still showing polar GFP/LtrA localization, 39.5% showing partially or completely diffuse fluorescence, and 16.9% filamentous cells with multiple foci and/or irregular diffuse patches of GFP/LtrA fluorescence (Figure 2 and Table 1, top). The *wcaK* disruptant also showed an increased proportion of filamentous cells (18.6%), while the remaining three disruptants showed few or no filamentous cells. The ratio of cells with completely or partially diffuse LtrA localization differed for each of the disruptants, being highest for the *zntR* disruptant and lowest for the *uhpT* disruptant. When Ll.LtrB intron expression was induced under the higher temperature conditions (overnight, 100 μM IPTG, 37 °C), we observed similar GFP/LtrA localization patterns, but the proportions of cells showing completely or partially diffuse fluorescence shifted in some cases (Table S1).

Although their cellular phenotypes are heterogeneous, PCR using primers flanking the target gene showed that cultures of four of the disruptants (*gppA*, *uhpT*, *wcaK*, and *ynbC*) were homogenous for the disrupted allele, while the fifth (*zntR*) contained predominantly the disrupted allele but also showed a light band (star) comigrating with that for the wild-type allele (Figure S4). This light band was found by sequencing to contain a 4-bp insertion at the transposon-insertion site in the middle of the ORF and is presumably a null allele resulting from transposon excision. Thus, in all cases, the heterogeneous cellular phenotype is not due to persistence of the wild-type allele.

### Intron Mobility Frequencies in the Disruptants

**Figure 3:**
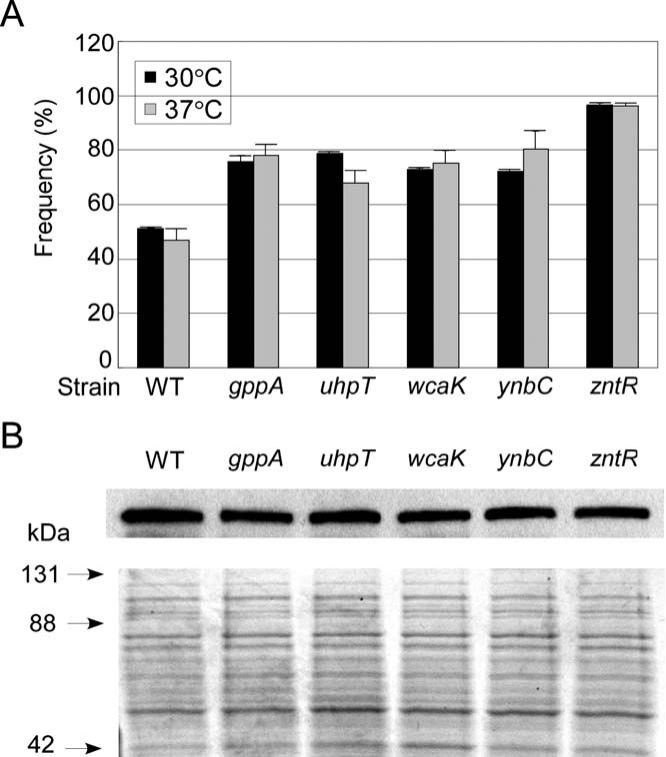
Ll.LtrB-ΔORF Intron Mobility Frequencies in Wild Type and Disruptants with Altered GFP/LtrA Localization Patterns (A) Mobility frequencies. Donor plasmid pACD2X containing an Ll.LtrB-ΔORF intron retargeted to insert at a site in the *E. coli lacZ* gene (between nucleotide residues 1062 and 1063, antisense strand; Figure S5) was transformed into the indicated strains and induced with 500 μM IPTG for 0.5 h at 30 °C (black bars) or 100 μM IPTG for 1 h at 37 °C (gray bars). The cells were then plated on LB medium containing X-Gal, and the *lacZ* integration frequency was calculated as the percentage of white colonies. The bar graphs show the mean for three independent assays, with the error bars indicating the standard deviation. (B) LtrA expression levels in the cultures used for mobility assays after induction with IPTG at 30 °C. Top, immunoblot probed with an anti-LtrA antibody preparation. Bottom, a parallel gel stained with Coomassie blue. Arrows to the left of the gel indicate positions of size markers (Kaleidoscope Prestained Standard; Bio-Rad).

Next, we tested whether the disruptions affect intron mobility frequencies. For these experiments, we used an Ll.LtrB-ΔORF intron that had been retargeted by modification of its EBS and δ sequences to insert at a site within the *E. coli lacZ* gene (LacZ-1063a) so that the intron integration frequency could be scored simply by blue–white screening (see Materials and Methods section and Figure S5). Intron-donor plasmid pACD2X expressing the retargeted intron was transformed into the wild-type and disruptant strains and induced with IPTG at 30 or 37 °C prior to plating the cells on Luria–Bertani (LB) medium containing X-Gal. As summarized in Figure 3, at both temperatures, Ll.LtrB-ΔORF intron mobility frequencies in the disruptants were somewhat higher than those in the wild-type strain (68–96% compared to 47–51%). Immunoblots of proteins isolated from the induced cells showed slightly reduced levels of LtrA protein in all cases (69–78% wild type; Figure 3B), indicating that the increased mobility frequencies simply are not due to higher protein expression levels. Possible reasons for the increased chromosomal insertion frequencies in the disruptants are discussed below (see Discussion section).

### Chromosomal Distribution of Ll.LtrB Intron-Insertion Sites in Wild-Type and Disruptant Strains

Having demonstrated that the Ll.LtrB-ΔORF intron remains mobile in the disruptants, we next examined whether the changes in LtrA localization patterns are correlated with changes in the genomic distribution of Ll.LtrB-insertion sites. As done previously to analyze insertion-site preference [10], we used a donor plasmid library that expresses Ll.LtrB-ΔORF introns with randomized target site recognition (EBS2, EBS1, and δ) sequences plus a Tp^R^ retrotransposition-activated marker (RAM) to detect chromosome integrations. This marker consists of a small trimethoprim-resistance (*Tp^R^*) gene inserted within the intron in the orientation opposite to intron transcription but interrupted by a small, self-splicing group I intron (the phage T4 *td* intron) in the forward orientation. During retrohoming via an RNA intermediate, the group I intron is spliced, reconstituting the marker and enabling the selection of cells containing integrated introns by trimethoprim resistance. The Tp^R^ colonies were isolated and analyzed by TAIL PCR and sequencing to identify the intron-insertion sites.

**Figure 4:**
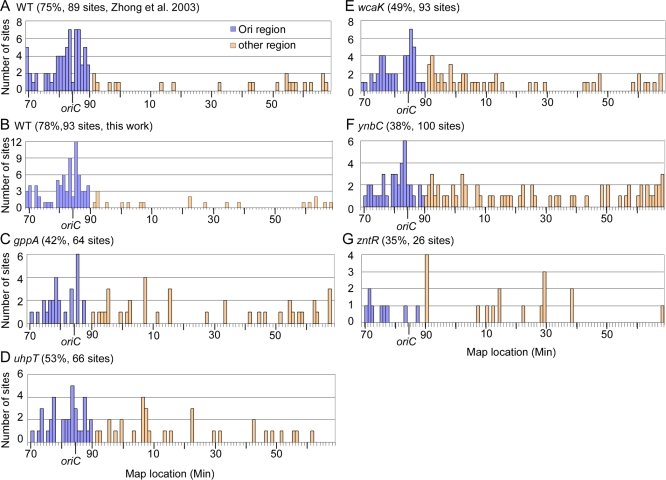
Ll.LtrB-ΔORF Intron Insertion Site Distribution in Wild Type and Disruptants with Altered GFP/LtrA Localization Patterns Wild-type HMS174(DE3) and disruptants showing altered GFP/LtrA localization patterns were transformed with pACD3-Tp^R^-RAM containing a library of Ll.LtrB-ΔORF introns with randomized EBS1, EBS2, and δ sequences plus a Tp^R^-RAM in intron domain IV. After induction, cells were plated on medium containing trimethoprim to select cells containing integrated Ll.LtrB introns carrying the activated Tp^R^-RAM, and insertion sites in randomly selected colonies were identified by TAIL PCR and sequencing. (A and B) Chromosomal distribution of Ll.LtrB-ΔORF intron-insertion sites in wild-type E. coli HMS174(DE3) (WT) from Zhong et al. [10] and this work, respectively. (C–G) Chromosomal distribution of Ll.LtrB-ΔORF intron-insertion sites in the indicated disruptants with more diffuse GFP/LtrA localization patterns. Blue bars represent insertion sites within the 69–90 minute region encompassing *oriC* (21% of the genome), and peach bars represent insertion sites outside this region. The numbers above the graphs show the percentage of sites in the *oriC* region and the total number of independent sites sequenced for each strain. In two separate inductions with the *zntR* disruptant, a single but different insertion site outside the *oriC* region was found repeatedly among sequenced colonies (21/26 at 1325864 in *btuR* and 21/50 at 4199340 in *zraP*), resulting in a smaller number of independent sites sequenced for that strain. Such a repeated occurrence could reflect either that hot spots exist for intron integration or that the integration occurred prior to or early in induction and was amplified in the culture by cell division. In the other strains, two to three insertion sites, which differed in each strain, were repeated two to four times among sequenced colonies. In all cases, such repeated sites were counted as a single site in the analysis shown. If the repeated sites were counted as independent events, then the proportions in the *oriC* region would be WT, 79%; *gppA*, 45%; *uhpT*, 47%; *wcaK*, 51%; *ynbC*, 31%; and *zntR*; 14%.

The chromosomal distributions of Ll.LtrB-ΔORF intron-insertion sites in wild-type HMS174(DE3) and the disruptants are shown in Figure 4, with the previous distribution for wild-type HMS174(DE3) from Zhong et al. [10] shown for comparison. The two sets of data for the wild-type strain are in close agreement, showing Ll.LtrB intron-insertion sites strongly clustered around *oriC* (blue bars; Figure 4A and 4B). By combining the two data sets for the wild-type strain, we defined the region around *oriC* containing clustered Ll.LtrB-insertion sites as encompassing minutes 69–90 of the E. coli chromosome (21% of the genome).

In the wild-type strain, the proportion of Ll.LtrB-insertion sites in the *oriC* region defined as above was 75% in the previous work (Figure 4A) and 78% in the present work (Figure 4B), strikingly higher than 21% expected for random integration. By contrast, in the disruptants only 35–53% of the Ll.LtrB-insertions sites were located in the *oriC* region, with the lowest proportions being found in the *zntR* and *ynbC* disruptants (35% and 38%, respectively; Figure 4C–G). The differences in distribution patterns were statistically significant at *p* < 0.001, calculated by χ-square test. These findings show that the more diffuse intracellular localization of GFP/LtrA in the disruptants is in fact correlated with a more uniform distribution of Ll.LtrB-insertion sites.

### Clues from the *gppA* Disruptant: GFP/LtrA Localization Is Not Affected by Decreased Synthesis of Magic Spot, but Is Affected by Polyphosphate Accumulation

Among the genes whose disruption results in altered GFP/LtrA localization, the best characterized is *gppA*, which encodes guanosine pentaphosphatase A (GPPA). This enzyme has two functions: it dephosphorylates pppGpp to produce ppGpp (“magic spot”), and it processively hydrolyzes intracellular poly(P), liberating orthophosphate [18].

**Figure 5:**
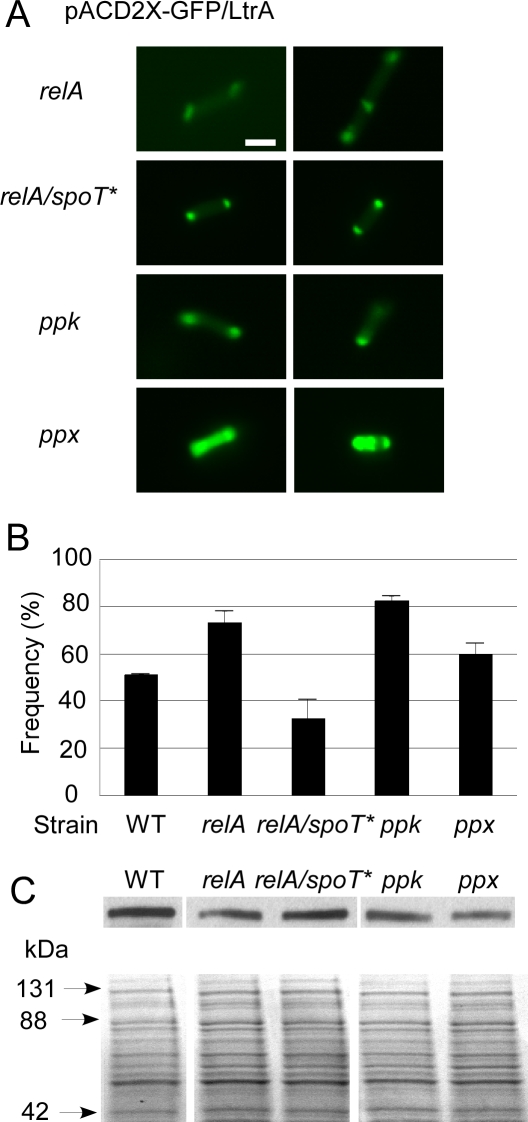
GFP/LtrA Localization Patterns and Intron Mobility Frequencies in Mutants Affecting Magic Spot and Poly(P) Accumulation (A) Fluorescence microscopy of GFP/LtrA localization in a *relA* disruptant and a *relA*/spoT* mutant defective in synthesis of magic spot and in *ppk* and *ppx* disruptants, which inhibit the synthesis and degradation of poly(P), respectively. Cells containing pACD2X-GFP/LtrA were induced with 500 μM IPTG overnight at 30 °C. For each strain, ≥200 cells were examined by fluorescence microscopy, and the proportions of cells with different LtrA localization patterns are summarized in Table 1, bottom. The localization patterns shown in the figure represent the most common pattern for each strain. Bar = 2 μm. (B) Mobility frequencies of the Ll.LtrB-ΔORF intron in different strains determined by using a chromosomal *lacZ* gene disruption assay (see Figure 3 and Materials and Methods section). The bar graphs show the mean for three independent assays, with the error bars indicating the standard deviation. (C) LtrA expression levels from cultures used in the mobility assay. Top, an immunoblot probed with an anti-LtrA antibody preparation. Bottom, a parallel gel stained with Coomassie blue (all lanes from the same gel). Arrows to the left of the gel indicate positions of size markers (Kaleidoscope Prestained Standard; Bio-Rad). Asterisks indicate that the strain was obtained from GenoBase.

To test whether the altered LtrA localization pattern in the *gppA* disruptant is due to impaired synthesis of magic spot, we examined LtrA localization in two other mutants that affect the synthesis of magic spot in different ways: a *relA* disruptant derived from wild-type HMS174(DE3) by targetron mutagenesis (Figure S5) and a previously isolated *relA*/spoT* double mutant obtained from GenoBase [24]. (Note that strains obtained from GenoBase were derivatized with λDE3 for intron expression and are denoted with an asterisk to indicate different genetic background.) Both the *relA* and the *relA*/spoT* mutants still showed predominantly polar localization of GFP/LtrA (72.2% and 69.2% of cells, respectively), with only small proportions of cells (4.2% and 6.3%, respectively) showing diffuse GFP/LtrA localization (Figure 5A and Table 1, bottom). Thus, impaired ability to produce ppGpp does not strongly affect LtrA localization. The mobility frequency of the Ll.LtrB-ΔORF intron assayed by targeted integration into the *lacZ* gene as above was increased in the *relA* disruptant and decreased in the *relA*/spoT* mutant compared to the wild-type strain (Figure 5B). The lower intron mobility frequency in the *relA*/spoT* mutant could be due to the *relA*/*spoT* mutations, the different genetic background, or a combination of the two.

The alternate possibility was that GFP/LtrA localization in the *gppA* disruptant might be due to the accumulation of intracellular poly(P). To test this possibility, we used targetron mutagenesis to disrupt the E. coli HMS174(DE3) *ppk* and *ppx* genes, which encode polyphosphate kinase and exopolyphosphatase, respectively [25,26] (Figure S5). The *ppk* and *ppx* genes are expressed from the same operon, with *ppk* upstream of *ppx*. Thus, the disruption of *ppk* is expected to affect the expression of both genes and lead to decreased levels of intracellular poly(P), while the disruption of *ppx* should affect only the expression of that gene and lead to accumulation of poly(P) [26,27].

The *ppx* disruptant, which we confirmed below accumulates poly(P), did in fact show a substantially increased proportion of cells with more diffuse GFP/LtrA localization (27.4%), while the *ppk* disruptant, expected to have decreased levels of poly(P), showed predominantly the normal bipolar GFP/LtrA localization pattern (Figure 5A and Table 1, bottom). Similar results were obtained with independently constructed *ppx* and *ppk* deletions from the Keio collection obtained from GenoBase (i.e., more diffuse GFP/LtrA localization in the ppx* deletion (18.7%) and predominantly bipolar GFP/LtrA localization in the ppk* deletion (Table 1, bottom)). In both the *ppk* and the *ppx* disruptants, the mobility frequency of the Ll.LtrB-ΔORF intron assayed by targeted integration into the *lacZ* gene was increased relative to the wild-type strain, with the increases more pronounced if normalized to the LtrA expression level (Figure 5B and 5C). We conclude from these findings that the common feature correlated with the more diffuse LtrA localization in the *gppA* and *ppx* disruptants is the accumulation of intracellular poly(P).

### Intracellular Distribution of Poly(P) in Wild-Type and Disruptant Strains

To investigate further the relationship between poly(P) accumulation and GFP/LtrA localization, we used fluorescence microcopy to examine the intracellular localization of poly(P) detected by 4′,6-diamidino-2-phenylindole (DAPI) staining in the wild-type strain and the *gppA* and *ppx* disruptants expressing GFP/LtrA. Under fluorescence microscopy with excitation at 360 nm, DAPI bound to poly(P) emits at 550 nm and appears yellow or orange, while DAPI bound to DNA emits at 490 nm and appears blue [28].

**Figure 6:**
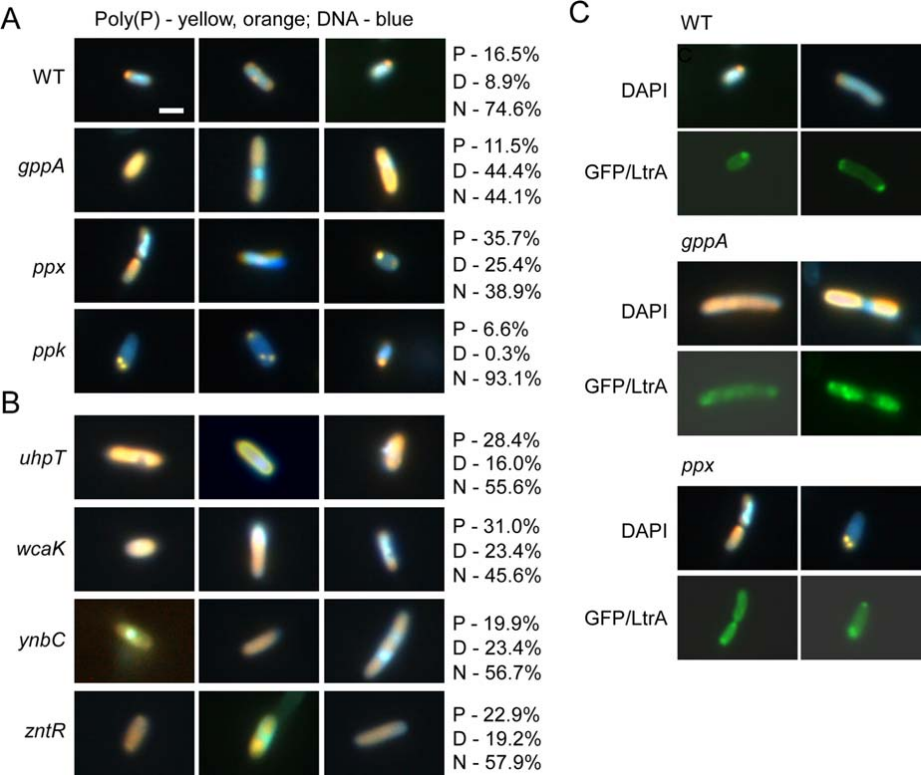
Fluorescence Microscopy of Poly(P) and GFP/LtrA Localization in Wild Type and Disruptants Wild-type HMS174(DE3) (WT) and disruptants containing pACD2X-GFP/LtrA were induced with 500 μM IPTG overnight at 30 °C, stained with DAPI, and examined by fluorescence microscopy. In cells stained with DAPI, poly(P) appears yellow or orange, and DNA appears blue. At least 200 randomly selected cells were analyzed in each case. Proportions of cells with normal morphology showing polar (P), diffuse (D), or no detectable fluorescence above background (N) are indicated to the right. (A) Poly(P) localization in WT, *gppA*, *ppx*, and *ppk* disruptants. (B) Poly(P) localization in *uhpT*, *wcaK*, *ynbC*, and *zntR* disruptants. (C) Selected examples of cells showing poly(P) and GFP/LtrA colocalization. Bar = 2 μm.

In wild-type HMS174(DE3) under standard GFP/LtrA induction conditions at 30 °C, 16.5% of the cells showed detectable poly(P) fluorescence localized in discrete foci, mainly at the cell poles (P), another 8.9% showed more diffuse poly(P) fluorescence (D), and the remainder (74.6%) showed no detectable poly(P) fluorescence (N), likely reflecting at least in part the sensitivity of the detection method (Figure 6A). The localization of poly(P) in discrete foci (“volutin granules”) at the cellular poles has been found previously in other bacteria [29–31].

By contrast, under the same conditions, both the *gppA* and the *ppx* disruptions, which lack enzymes involved in poly(P) degradation, showed substantially increased proportions of cells with detectable poly(P) fluorescence (55.9% and 61.1%, respectively; Figure 6A). Further, in a high proportion of *gppA* and *ppx* disruptant cells, poly(P) was no longer present in discrete foci but was instead dispersed throughout the cell (44.4% and 25.4%, respectively, excluding filamentous *gppA* cells). Conversely, the *ppk* disruptant, which lacks the major poly(P) biosynthetic enzyme, shows a reduced proportion of cells with detectable poly(P) fluorescence, which was localized again mainly at or near the cell poles (6.6%; Figure 6A). The residual poly(P) in the *ppk* disruptant may be synthesized by an alternate pathway [32]. Similar differences in poly(P) localization between the wild-type and the disruptant strains were observed after IPTG induction in strains carrying the empty vector, indicating that poly(P) accumulation in the disruptants is not due to expression of GFP/LtrA (unpublished data).

Importantly, although the *gppA* and *ppx* disruptants have heterogeneous cellular phenotypes, poly(P) accumulation and dispersed GFP/LtrA localization were correlated strongly in individual cells. Thus, for the *gppA* disruptant, 90% of the doubly fluorescent cells with diffuse poly(P) localization showed diffuse GFP/LtrA localization, and 96% with diffuse GFP/LtrA localization showed diffuse poly(P) fluorescence. Similarly, in the *ppx* disruptant, 89% of the doubly fluorescent cells with diffuse poly(P) localization showed diffuse GFP/LtrA localization, and 88% with diffuse GFP/LtrA localization showed diffuse poly(P) localization. Further, in a high proportion of the doubly fluorescent cells, the poly(P) and GFP/LtrA fluorescence were either overlapping (94.7% and 87.9% in the *gppA* and *ppx* disruptants, respectively) or completely colocalized (78.4% and 45.5% in the *gppA* and *ppx* disruptants, respectively; Figure 6C). This degree of colocalization supports the hypothesis that the altered GFP/LtrA localization is due to the binding of the basic LtrA protein to the negatively charged poly(P). Those cells in which both poly(P) and GFP/LtrA are dispersed but not completely colocalized could reflect that poly(P) associated with LtrA dissociates or is degraded after the protein is dispersed, that some poly(P) bound to LtrA is less than the concentration limit required for fluorescence detection with DAPI, or that the GFP tag is clipped, rendering some proportion of LtrA nonfluorescent.

After the above findings, we also re-examined the wild-type strain, where 2.8% of the cells showed diffuse GFP/LtrA localization (Table 1, top). Strikingly, even in wild type, where only a small proportion of cells is affected, diffuse GFP/LtrA localization was correlated again with the accumulation and dispersal of poly(P) (88% of wild-type cells with diffuse GFP/LtrA showed dispersed poly(P), and 83% with dispersed poly(P) showed diffuse GFP/LtrA). The accumulation of poly(P) in a small proportion of wild-type cells is likely due to cell stress, which triggers poly(P) synthesis [33]. Collectively, these findings suggest that the more diffuse intracellular localization of GFP/LtrA results from the accumulation of poly(P), which binds to LtrA and delocalizes it away from the cell poles.

### All the Other Disruptants with Diffuse GFP/LtrA Localization Also Accumulate Poly(P)

The remaining four disruptants (*uhpT*, *wcaK*, *ynbC*, and *zntR*) with more diffuse GFP/LtrA localization do not involve genes that are known to function in poly(P) metabolism. However, the above findings raised the possibility that they might also accumulate poly(P) as a result of cell stress caused by the disruptions. Fluorescence microscopy of DAPI-stained cells revealed that this is in fact the case, with all four of the above disruptants in the HMS174(DE3) genetic background showing an increased proportion of cells with higher and more dispersed poly(P) fluorescence (Figure 6B). By contrast, strains having deletions of the same four genes (uhpT*, wcaK*, ynbC*, and zntR*) in the Keio genetic background did not accumulate poly(P) and correspondingly did not exhibit altered GFP/LtrA localization patterns (unpublished data). The likely explanation is that their different genetic background makes the Keio strains less prone to cell stress caused by these mutations and/or less prone to accumulate poly(P) in response to cell stress than in the HMS174(DE3) background. In comparison to the *ppx* disruption in the HMS174(DE3) background (Figure 6), the ppx* deletion in the Keio genetic background also showed less detectable poly(P) accumulation (1.0% P, 6.0% D, 93.0% N) and correspondingly had a more muted effect on GFP/LtrA localization (Table 1, bottom). We conclude that poly(P) accumulation is the common factor correlated with altered GFP/LtrA localization in all of the strains analyzed here.

### Poly(P) Binds to LtrA and Inhibits Its Reverse Transcriptase Activity

**Figure 7:**
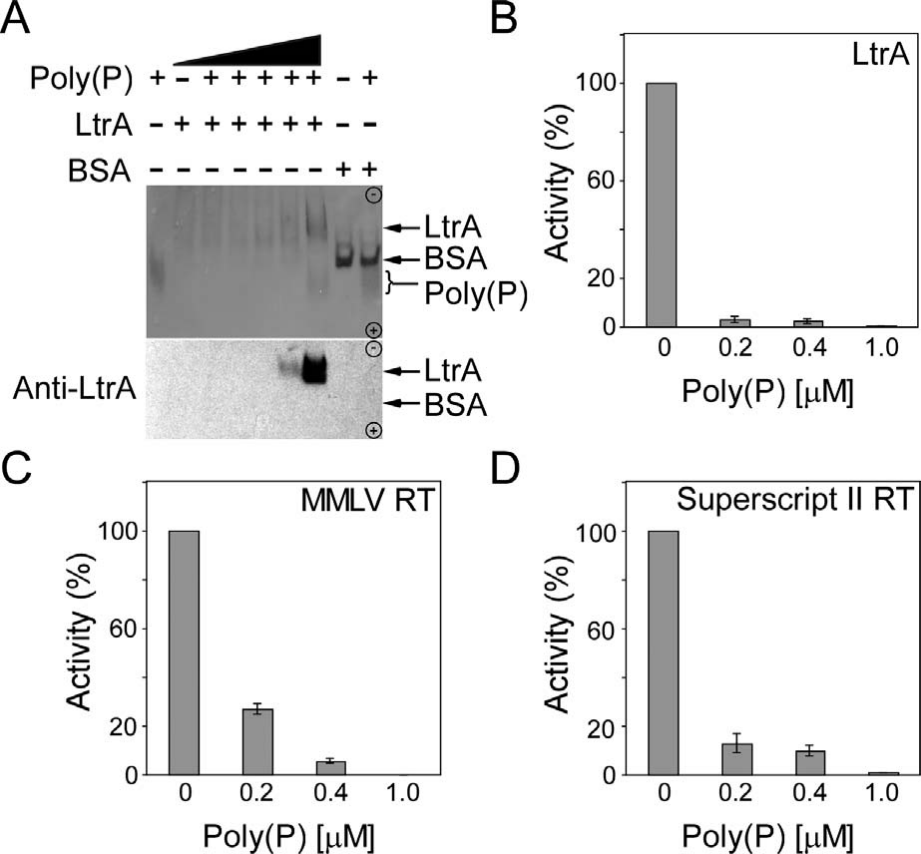
Poly(P) Binds to LtrA and Inhibits its RT Activity (A) Nondenaturing PAGE. LtrA protein (100 pmol) by itself or mixed with different amounts of poly(P) (1 pmol, 10 pmol, 100 pmol, 1 nmol, and 10 nmol) was run in a nondenaturing 5–15% polyacrylamide gradient gel. Bovine serum albumin (100 pmol; BSA) by itself or mixed with10 nmol poly(P) was run in parallel lanes. The gel was silver-stained, and a parallel gel (below) was blotted to nitrocellulose and probed with anti-LtrA antibody (see Materials and Methods section). (B–D) RT assays. RT activity was assayed by incubating 200 nM LtrA or dilutions of Moloney murine leukemia virus or Superscript II RT (Invitrogen) having equivalent activity with [^32^P]-dTTP and poly(rA)/oligo(dT)_18_ in reaction medium containing 10 mM KCl, 10 mM MgCl_2_, and 50 mM Tris-HCl, pH 7.5, for 10 min at 37 °C in the presence or absence of 0.2, 0.4, or 1 μM poly(P). Polymerization of [^32^P]-dTTP was measured as described in the Materials and Methods section and [50]. For all three enzymes, RT activities were 20,000–100,000 cpm, and incubation times were verified to be in the linear range. The bar graphs show the mean activity for three independent determinations, with the error bars indicating the standard deviation.

To test directly whether poly(P) binds to LtrA, we examined its effect on the electrophoretic mobility of the LtrA protein in a nondenaturing polyacrylamide gel (Figure 7A). In the absence of poly(P), the LtrA protein, which is highly positively charged (calculated pI = 9.6), could not migrate toward the positive pole and failed to enter the gel. By contrast, with increasing concentrations of poly(P), an increasing proportion of LtrA entered the gel and migrated toward the positive pole, indicating complex formation. Control lanes (right) show that the highest concentration of poly(P) tested had no effect on the electrophoretic mobility of an acidic protein, bovine serum albumin (pI = 4.6), run in the same gel. Figure 7B extends these findings by showing that equimolar poly(P) completely inhibited the reverse transcriptase (RT) activity of LtrA. Poly(P) also inhibited the RT activity of Moloney murine leukemia virus and Superscript RTs, although these enzymes appeared somewhat less sensitive to poly(P) inhibition than LtrA (Figure 7C and 7D). Together, the above findings show that poly(P) binds LtrA and inhibits its RT activity. The ability of poly(P) to bind LtrA and carry it toward the opposite pole in a nondenaturing gel supports its hypothesized mechanism of action in the cell.

### The *gppA* and *ppx* Disruptants Show Altered Localization of Other Basic Proteins

**Figure 8:**
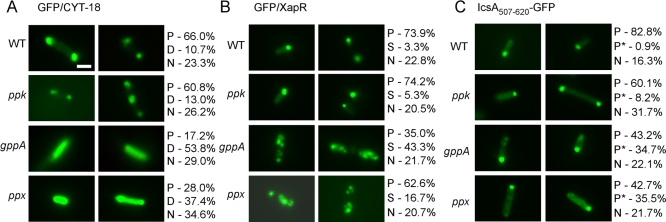
Disruptants that Accumulate Poly(P) Show Altered Intracellular Localization of Other Basic Proteins Fluorescence microscopy of wild-type HMS174(DE3) (WT) and the indicated disruptants containing (A) pAC-GFP/CYT-18, (B) pAC-GFP/XapR, or (C) pBAD24-*icsA*_507–620_ *::gfp*. Cells were induced with (A and B) 500 μM IPTG or (C) 0.2% l-arabinose overnight at 30 °C. At least 200 cells were examined in each case, and the proportions of cells showing polar (P), diffuse (D), scattered (S), polar and dispersed tiny foci (P*), or no detectable poly(P) fluorescence above background (N) are indicated to the right. The localization patterns shown in the figure represent the most common pattern for each strain. Bar = 2 μm.

Finally, we used the *gppA* and *ppx* disruptants to test whether poly(P) accumulation might similarly affect the polar localization of proteins other than LtrA. First, we tested GFP fusions of two basic proteins, which were shown previously to be pole-localized in E. coli: the N. crassa CYT-18 protein (calculated pI = 9.29) [12] and the E. coli transcriptional regulator XapR (calculated pI = 8.91) [34]. Strikingly, we found that both proteins are largely pole-localized in wild type and the *ppk* disruptant but showed more dispersed localization patterns in the *gppA* and *ppx* disruptants, which accumulate poly(P) (Figure 8A and 8B). For GFP/CYT-18, the *gppA* and *ppx* disruptants showed high proportions of cells with more diffuse protein localization, while for GFP/XapR these disruptants showed high proportions of cells with multiple large foci scattered throughout the cell. For GFP/XapR, we confirmed by dual-fluorescence microscopy that the scattered localization in individual cells is again correlated with the accumulation and dispersal of poly(P) (86% of the *gppA* and 83% of the *ppx* cells showing scattered GFP/XapR localization also showed dispersed poly(P) localization). Immunoblots showed that GFP/XapR is expressed at similar levels in the wild-type and *gppA* and *ppx* disruptant strains (Figure S6).

By contrast to these basic proteins, a GFP fusion with a S. flexneri IcsA protein subsegment (IcsA_507–620_ pI = 7.15) [35] remained pole-localized in the *gppA* and *ppx* disruptants, although for both mutants an increased proportion of cells with pole-localized IcsA_507–620_/GFP showed additional tiny foci distributed throughout the cell (P*; Figure 8C). Thus, as expected, poly(P) accumulation most strongly affects the localization of positively charged proteins to which it can bind directly.

## Discussion

Here, by using high-throughput methods to screen a transposon-insertion library, we identified five E. coli genes (*gppA*, *uhpT*, *wcaK*, *ynbC*, and *zntR*) whose disruption affects the polar localization of LtrA, a group II intron-encoded reverse transcriptase that functions with the intron RNA in RNPs to promote intron mobility. All of the above disruptants show both an increased proportion of cells with more diffuse GFP/LtrA localization and a more uniform genomic distribution of Ll.LtrB-insertion sites, which in the wild-type E. coli strain are strongly clustered in the *oriC* region. These findings indicate that the intracellular localization of LtrA is a major determinant of Ll.LtrB-insertion-site preference, to our knowledge the first such demonstration for any mobile genetic element. Further analysis showed that the common factor leading to more diffuse GFP/LtrA localization in these and other disruptants is the accumulation of intracellular poly(P) and that disruptants that accumulate poly(P) also show altered intracellular distributions of other basic proteins that are normally pole-localized (CYT-18 and XapR). The latter findings suggest that poly(P) accumulation may be part of a cellular mechanism that leads to relocalization of basic proteins in response to cell stress or entry into stationary phase. From a technical standpoint, our results demonstrate the feasibility of using bacterial cellular arrays for high-throughput screens to identify mutations affecting protein localization or morphology, and they suggest a method for obtaining more uniform group II intron-gene disruption libraries by using mutants that accumulate poly(P).

The polar localization of LtrA in wild-type cells may facilitate group II intron mobility by increasing access of group II intron RNPs to exposed DNA segments in the *oriC* and *ter* regions of the E. coli chromosome. It also may provide favorable sites for interaction with DNA replication components and a means of coordinating group II intron mobility with DNA replication and/or cell division [12]. Other mobile elements use a variety of mechanisms for coordinating transposition with DNA replication ([36,37] and references therein). Such coordination may be particularly important for group II introns, which can use nascent strands at DNA replication forks to prime reverse transcription [11,38,39]. Indeed, Coros et al. [11] found that in E. coli the frequency of Ll.LtrB retrotransposition events that use this priming mechanism is highest in the *oriC* domain and decreases in a gradient toward the *ter* domain, while retrotransposition events that utilize the DNA cleavage activity of the IEP to generate the primer for reverse transcription do not show such a gradient. The selection for polar localization may have originated with ancestral group II introns whose IEPs lacked DNA cleavage activity and relied entirely on nascent DNA strands as primers.

In addition to changing the intracellular localization of LtrA, poly(P) also potentially could affect the genomic distribution of Ll.LtrB-insertion sites by binding basic nucleoid-associated proteins, leading to nucleoid decondensation, as observed in a *Pseudomonas aeruginosa ppk1* mutant [40]. Indeed, it has been speculated that such a mechanism contributes to global changes in gene expression accompanying poly(P) accumulation in response to cell stress or entry into stationary phase [41]. However, Beauregard et al. [15] found that neither LtrA localization nor the distribution of Ll.LtrB-insertion sites is affected significantly by mutations in the nucleotide-associated proteins H-NS, StpA, or MukB, which lead to nucleoid decondensation in different ways. These findings indicate that nucleoid decondensation does not by itself lead to a more uniform distribution of Ll.LtrB-insertion sites as long as LtrA remains pole-localized. It is possible, however, that nucleotide decondensation contributes to the more uniform distribution of insertion sites that we observe when LtrA has been delocalized by poly(P).

Our results suggest that negatively charged poly(P) delocalizes LtrA and other basic proteins by binding to them directly. A direct binding mechanism is supported by: (i) the colocalization of GFP/LtrA and poly(P) in high proportions of *gppA* and *ppx* disruptant cells (Figure 6C); (ii) the finding that poly(P) accumulation affects the polar localization of three basic proteins (LtrA, CYT-18, and XapR) but not the polar localization of the acidic protein IcsA_507–620_/GFP (Figure 8); and (iii) biochemical experiments showing that poly(P) can bind LtrA to inhibit its RT activity and cause it to migrate toward the opposite pole in a nondenaturing polyacrylamide gel (Figure 7). The binding of poly(P) to LtrA and other basic proteins is presumably nonspecific, but we cannot at this stage exclude a more specific binding component in some cases. In the cell, poly(P) may bind to LtrA and other basic proteins at the poles and relocalize them as it moves through the cell, and/or it may bind to the nascent proteins in other regions, preventing them from becoming pole-localized. The poly(P) that colocalizes with LtrA could be present in specific complexes, akin to volutin granules, which may be passively or actively localized or delocalized.

In addition to affecting the intracellular localization of LtrA, the binding of poly(P) may directly affect LtrA's biochemical activities in RNA splicing and intron mobility. Precedents for such effects include the findings that poly(P) activates Lon protease degradation of ribosomal proteins [42], may play a role in activating mammalian TOR kinase [43], and is required for the lytic growth of phages P1 and fd [44]. Our finding that poly(P) inhibits LtrA's RT activity (Figure 7B) may explain why the *ppk* mutant, which has decreased levels of poly(P), shows increased Ll.LtrB mobility frequencies in a chromosomal *lacZ* gene integration assay (Figure 5B). However, we also find that Ll.LtrB mobility frequencies are increased moderately in *gppA*, *ppx*, and other mutants that accumulate poly(P) (Figures 3A and 5B). The latter findings indicate that inhibition of LtrA's RT activity by poly(P) in vivo must be either mild or transient, perhaps due to dissociation or degradation of the bound poly(P) after the protein has been delocalized. A factor that may contribute to the moderately increased Ll.LtrB mobility frequencies in mutants that accumulate poly(P) is that their more uniform intracellular distribution of LtrA makes it easier to target the *lacZ* gene, which is located outside the *oriC* or *ter* chromosomal regions.

The disruptants that we identified with altered GFP/LtrA localization patterns accumulate poly(P) for different reasons. The *gppA* and *ppx* disruptions inhibit poly(P) degradation, while the *uhpT*, *wcaK*, *ynbC*, and *zntR* disruptants presumably accumulate poly(P) as a result of cellular stress caused by the disruptions. All of disruptants, including the *gppA* and *ppx* disruptants, have heterogeneous cellular phenotypes, with an increased proportion of cells showing both poly(P) accumulation and altered GFP/LtrA localization, and the remainder having wild-type localization patterns. This heterogeneity may reflect that only some cells in the population are under sufficient stress to trigger poly(P) accumulation. The wild-type strain shows similar heterogeneity but with only a small proportion of cells showing poly(P) accumulation and altered GFP/LtrA localization. Thus, the disruptions appear to increase the normal propensity for stress-induced poly(P) accumulation, either by increasing the degree of stress or by decreasing the degradation rate of poly(P), making it easier to achieve elevated poly(P) concentrations. We note that the *gppA* disruption causes a more extreme accumulation and dispersal of poly(P) than does the *ppx* disruptant (Figure 6), possibly reflecting that the *gppA* disruptant not only lacks GPPA, which contributes to poly(P) degradation, but also accumulates pppGpp, which inhibits the remaining exopolyphosphatase PPX [42].

Like a previous screen for E. coli localization factors [16], our cell array screen did not identify a pole-localized receptor protein whose absence leads to diffuse LtrA localization. It is possible that a specific polar receptor for LtrA does not exist and that LtrA is localized to the poles by default because it is not actively localized elsewhere. Alternatively, the receptor may require essential proteins or be redundant or nonspecific (e.g., acidic phospholipids [45]). Additionally, the intriguing finding that in wild-type E. coli poly(P) detected by DAPI staining is found frequently in discrete foci at the poles raises the possibility that poly(P) itself contributes as a receptor for the polar localization of basic proteins under nonstress conditions. The finding that GFP/LtrA and other basic proteins remain pole-localized in *ppk* mutants, which have ∼10-fold decreased poly(P) levels [27], does not exclude this possibility because poly(P) may be present in excess in wild-type strains and the residual poly(P) in the mutant may be sufficient for protein localization.

Finally, the finding that disruptants that accumulate poly(P) show altered distributions not only of LtrA but also of other pole-localized basic proteins suggests that poly(P) accumulation may be part of a mechanism that relocalizes proteins to different sites of action in response to cellular stress. One can imagine that reservoirs of certain enzymes, such as transcription factors or DNA repair enzymes, accumulate at the cellular poles and are mobilized to new sites of action by poly(P) during entry into stationary phase or under stress conditions, as shown here for LtrA. Such relocalization may be particularly advantageous when the cell's biosynthetic capacity is impaired as it is faster and more economical than synthesizing a specific receptor and transport system for each protein. Protein relocalization by binding to poly(P) may have wide physiological consequences, not only in prokaryotes but also in eukaryotes, where poly(P) also exists but has remained enigmatic.

## Materials and Methods

### Bacterial strains and growth conditions.

E. coli HMS174(DE3) (F^–^ *recA hsdR rif^R^*) (Novagen) was used for LtrA localization and Ll.LtrB intron-integration assays; DH5α was used for cloning; and S17–1λ*pir* [46] (obtained from Ram Narayanaswamy and Andy Ellington, University of Texas at Austin) was used for *mariner* transposon mutagenesis. Derivatives of HMS174(DE3) with disruptions of the *ppk*, *ppx*, and *relA* genes were constructed by targetron mutagenesis, as described ([8]; Figure S5). Keio deletion strains obtained from GenoBase (http://ecoli.naist.jp/GB6/search.jsp) were *ppk*, *ppx*, *uhpT*, *wcaK*, *ynbC*, and *zntR*. Other mutants obtained from GenoBase were *gppA* and *relA/spoT*. GenoBase strains were lysogenized with λ(DE3) carrying an IPTG-inducible T7 RNA polymerase gene by using a kit (Novagen) and are indicated with an asterisk in the text to denote their different genetic background. Strains were grown in LB medium at 30 or 37 °C, with antibiotics used at the following concentrations: ampicillin, 100 μg/ml; chloramphenicol, 25 μg/ml; kanamycin, 40 μg/ml; rifampicin, 100 μg/ml; trimethoprim, 10 μg/ml.

### Recombinant plasmids.

pACD2X-GFP/LtrA expresses the Ll.LtrB-ΔORF intron and a GFP/LtrA fusion protein (Figure 1A) [12]. The pACD3-Tp^R^-RAM library expresses Ll.LtrB-ΔORF introns with randomized EBS2, EBS1, and δ sequences plus a Tp^R^-RAM for detecting chromosome integrations [10]. Plasmids expressing other GFP fusion proteins were: pAC-GFP/CYT-18, N. crassa CYT-18 protein with an N-terminal GFP fusion [12]; pBAD24-*icsA*_507–620_ *::gfp*, pole-localized segment of the S. flexneri IcsA protein with a C-terminal GFP fusion [35]; and pAC-GFP/XapR, E. coli XapR protein with an N-terminal GFP fusion. The latter plasmid was derived from pAC-GFP/LtrA [12] by replacing the LtrA ORF with the *xapR* ORF (codons 1–294) amplified from E. coli HMS174(DE3) by PCR. pSC189 expresses a *mariner* transposon with a *kan^R^* marker and a separately encoded hyperactive C9 transposase [47]. The protein expression plasmid pImp-1P contains the LtrA ORF cloned behind a *tac* promoter in pCYB2 (New England Biolabs) [4].

### Construction of a *mariner* transposon-insertion library.

E. coli strains HMS174(DE3) and S17–1λ*pir* carrying pSC189 (see above) were grown separately to OD_600_ = 0.5 at 37 °C in 10 ml of LB without and with ampicillin, respectively, then mixed, and incubated at room temperature overnight without shaking for conjugation. The conjugated cells were washed twice with LB medium by centrifugation, resuspended, and grown overnight at 37 °C in fresh LB medium containing rifampicin and kanamycin to kill the donor strain and select for recipient HMS174(DE3) cells containing *mariner* transposon insertions. Loss of pSC189, which carries an *amp^R^* marker and does not replicate in HMS174(DE3), was confirmed by plating on LB agar with and without ampicillin (<1% Amp^R^ colonies). The HMS174(DE3) isolate used for library construction inadvertently carried a Tet^R^ broad-host-range plasmid pBBR1MCS-3 [48]. For the GFP/LtrA localization screen, cells grown in LB with kanamycin were electroporated with the intron-donor plasmid pACD2X-GFP/LtrA and plated on LB agar containing chloramphenicol and kanamycin. Approximately 9,600 colonies were picked into one hundred 96-well plates and stored at −80 °C. TAIL PCR of isolates showed that >92% contained different *mariner* transposon insertions, both before and after introduction of pACD2X-GFP/LtrA.

### Cell microarray construction and imaging.

The E. coli HMS174(DE3) *mariner* transposon-insertion library carrying pACD2X-GFP/LtrA was inoculated into new 96-well plates containing LB medium with chloramphenicol plus kanamycin and incubated overnight at 37 °C. The cultures then were inoculated 1:10 into fresh medium plus 17% glycerol in a new 96-well plate and grown for 5 h at 37 °C. In one screen, cells in fifty-one 96-well plates were induced overnight with 100 μM IPTG at 37 °C, and in another screen, cells in forty-nine 96-well plates were induced overnight with 500 μM IPTG at 30 °C. Culture transfer and media additions were done by a Biomek FX Laboratory Automation Workstation (Beckman Coulter).

Cell microarrays (cell chips) were constructed as described [17]. Briefly, ∼5,000 knockouts plus a wild-type HMS174(DE3) control were printed onto poly-l-lysine-coated microscope slides using a custom-built DNA microarray-printing robot. In each experiment, ∼30 cell chips were made, of which two were used for imaging and the remainder were stored at −80 °C. Before being imaged, the cell chips were washed briefly with double-distilled water to remove glycerol and debris and then mounted with VECTASHIELD hard-set mounting medium containing 1.5 μg/ml DAPI. Cell images were collected by automated microscopy, using a Nikon E800 fluorescence microscope with computer-controlled *X-Y* stage and piezoelectric-positioned objective. The automated microscope scanned the position of each spot, focused, and captured the image with a Coolsnap CCD camera (Photometrics). Images were stored in a custom cell microarray image database (Cellma, http://cellma.icmb.utexas.edu/) and examined individually to identify strains with altered GFP/LtrA localization patterns.

### Fluorescence microscopy.

Fluorescence microscopy was done as described [12]. Cells carrying pACD2X-GFP/LtrA were grown in LB medium with appropriate antibiotics at 37 °C to OD_600_ = 0.3 and then induced overnight with 500 μM IPTG at 30 °C or 100 μM IPTG at 37 °C. DAPI (25 μg/ml) was added to cultures 30 min before the end of induction. Cells were examined by fluorescence microscopy using a Leica DMIRBE microscope (Leica) with a 100× oil lens (PL APO 1.4–0.7 NA) and a GFP filter for GFP fluorescence or a wide DAPI filter for poly(P) fluorescence. Photographs were taken with a Leica DFC350 FX fluorescence camera (GFP fluorescence) or a Leica DFC320 FX color camera (poly(P) fluorescence).

### Intron mobility assays.

Ll.LtrB-ΔORF intron mobility frequencies were determined by using a retargeted intron that inserts at a site within the *E. coli lacZ* gene (targetron LacZ-1063a; Figure S5). Cells containing the retargeted intron cloned in pACD2X were grown in LB medium with chloramphenicol at 37 °C to OD_600_ = 0.2–0.3 and induced with 500 μM IPTG for 0.5 h at 30 °C or 100 μM IPTG for 1 h at 37 °C. The cells then were washed with fresh LB medium and plated on LB containing X-Gal (Fisher Scientific). After overnight incubation at 37 °C, the *lacZ* integration frequency was calculated as the percentage of white colonies.

### Chromosomal distribution of Ll.LtrB-insertion sites.

Cells transformed with a pACD3-Tp^R^-RAM library of Ll.LtrB introns with randomized EBS2, EBS1, and δ sequences and a Tp^R^-RAM inserted in intron domain IV [10] were grown overnight at 37 °C in LB medium containing chloramphenicol (wild-type strains) or chloramphenicol plus kanamycin (disruptants), then inoculated 1:100 into fresh medium, grown to OD_600_ = 0.3, and induced with 500 μM IPTG overnight at 30 °C. Cells containing chromosomally integrated Ll.LtrB introns carrying the activated Tp^R^-RAM were selected by plating on Mueller–Hinton medium with trimethoprim and thymine [10].

### Thermal-asymmetric-interlaced PCR.

TAIL PCR [49] was done on colonies resuspended in PCR premix. Integration junctions were amplified by two successive PCRs, using two nested specific primers in combination with a single degenerate primer. For *mariner* transposon insertions, the first PCR used the specific primer TailP1 (5′-GTTCTTCTGAGCGGGACTCTGGGG-3′) and the degenerate primer AD2 (5′-NGTCGASWGANAWGA-3′, where N = A, C, G, or T; S = C or G; and W = A or T), and the second PCR used the specific primer TailP2 (5′-CGGCCGCGAAGTTCCTATTCCG-3′) and AD2. The final PCR product was gel-purified and sequenced using the TailP2 primer. For Ll.LtrB-intron insertions, the specific primers used in the first and second PCRs were Ell1 (5′-CTGATTAACATTGCGACTCAGTCGTACCC-3′) and Ell2 (5′-CAACCGTGCTCTGTTCCCGTATCAG-3′), respectively, and the degenerate primer was again AD2. The final PCR products were sequenced by using primer Ell3 (5′-GGTTGGCTGTTTTCTGTGTTATCTTACAGAG-3′).

### SDS-PAGE and immunoblotting.

Proteins isolated from equal amounts of cells (OD_600_= 0.02 or 0.04) were run in either a 7.5% polyacrylamide/0.1% SDS gel (GFP/LtrA, Figure S3) or a 10% polyacrylamide/0.1% SDS gel (LtrA, Figures 3 and 5; GFP/XapR, Figure S6), then transferred to a nitrocellulose membrane (Bio-Rad) by using a semidry transfer apparatus (Hoefer Semiphor TE 70; Amersham Biosciences). The blots were probed with a 1:5,000 dilution of anti-GFP antibody JL-8 (BD Biosciences), followed by a 1:10,000 dilution of horseradish peroxidase goat anti-mouse secondary antibody (Bio-Rad), or with a 1:1,000 dilution of an anti-LtrA antibody preparation (obtained from Gary Dunny, University of Minnesota), followed by a 1:100,000 dilution of horseradish peroxidase goat anti-rabbit secondary antibody (Pierce). Blots were developed with Amersham ECL western blotting detection reagents (GE Healthcare). Equal loading was confirmed by Coomassie blue staining of a parallel gel.

### Southern hybridization.

DNA was isolated from wild-type and mutants strains by using a genomic DNA isolation kit (Qiagen), digested with restriction enzymes, and run in a 0.8% agarose gel. The gel was blotted to a nylon transfer membrane (Magna, 0.45 μm; GE Osmonics Labstore) and hybridized with a ^32^P-labeled probe corresponding to *mariner* transposon positions 1385–1868. The probe was generated by PCR of pSC189 with primers Mar3200 (5′-GGGTGGAGAGGCTATTCGGCTATGACTGGGC-3′) and Mar-3650 (5′-CCTTGAGCCTGGCGAACAGTTCGGCTGG-3′), followed by labeling with [α-^32^P]dTTP (3,000 Ci/mmol; PerkinElmer), using a High Prime DNA labeling kit (Roche). The blots were scanned with a Typhoon Trio fluorescence scanner (Amersham Biosciences).

### Nondenaturing PAGE and RT assays.

The LtrA protein was expressed in E. coli Rosetta(DE3) (Novagen) from the intein-based expression plasmid pImp-1P (see above) and purified, essentially as described [4]. The LtrA protein used for RT activity assays was purified through an additional heparin-Sepharose chromatography step. For nondenaturing PAGE, poly(P) (Type 65; Sigma-Aldrich) was incubated with purified LtrA protein or bovine serum albumin (Fraction V (Sigma-Aldrich), with 20% glycerol added to match LtrA) in 20 μl of 100 mM KCl, 5 mM MgCl_2_, 20 mM Tris-HCl, pH. 7.5, for 30 min at 30 °C. The samples were then run in a nondenaturing 5–15% polyacrylamide gradient gel in Tris-acetate, pH 6.5, buffer. The gel was silver-stained, and a parallel gel was used for immunoblotting with anti-LtrA antibody (see above). RT activity was assayed as described [50] by polymerization of [^32^P]-dTTP (3,000 Ci/mmol; PerkinElmer) with poly(rA)/oligo(dT)_18_ as template in reaction medium containing 10 mM KCl, 10 mM MgCl_2_, and 50 mM Tris-HCl, pH 7.5, for 10 min at 37 °C. Samples were spotted onto DE-81 paper (Whatman), washed three times with 2× SSC (SSC is 0.15 M NaCl and 0.15 M citric acid, pH 7.0.), and counted in a scintillation counter.

## Supporting Information

Figure S1 Confirmation of *mariner* Transposon-Insertion Sites by PCR Analysis of the 5′- and 3′-Integration Junctions DNA isolated from the indicated disruptants by using a Genomic DNA Isolation Kit (Qiagen) was used as a template for PCR amplification of the 5′ (left) and 3′ (right) junctions of the inserted *mariner* transposon. The junction sequences were determined by sequencing the PCR products. The 5′ junction was amplified using primers P1 and P2, and the 3′ junction was amplified using primers P3 and P4. The gene-specific primers P1 and P4 were: *gppA*, gppA580 (5′-CAGTGTATGACCCTGGCGGGCGG-3′) and gppA-3end (5′-GCGTCAGCATCGCATCCGGCAC-3′); *uhpT*, uhpT1270 (5′-GGCTGGGCAGGCACCTTCGCCGCGC-3′) and ade1550 (5′-TGCCGTTACCCATTGCCGGGCTGATGAGC-3′); *wcaK*, wcaK50 (5′-GGGCAACCACACTTGCGGCAATCG-3′) and wcaK-350 (5′-CCTGATGCTGGTAGCGGCGACGGAGG-3′); *ynbC*, ynbC990 (5′-TTGTTCGGGACGCACTCTTCGGGCTGC-3′) and ynbC-1670 (5′-TCACGCACGAGTGAATCCATCTCCCC-3′); *zntR*, zntR-420 (5′-CCACTCTTAACGCCACTCGCCCCTTGTTC-3′) and yhdN30 (5′-GGCAGAGCGCCATATAGCAGAAGCGC-3′). The *mariner* primers P2 and P3 were: Mar-2520 (5′-GCTTCTCAGTGCGTTACATCCCTGGC-3′) and TAILP2 (5′-CGGCCGCGAAGTTCCTAT-TCCG-3′), respectively. The Figure shows PCR products run in a 1% agarose gel, which was stained with ethidium bromide. M, 1-kb DNA ladder (Invitrogen).
(5.2 MB TIF)

Figure S2 Confirmation of *mariner* Transposon-Insertion Sites by Southern Hybridization Genomic DNA (10 μg) from wild-type E. coli HMS174(DE3) (WT) and the indicated disruptants was digested with XcmI, XmaI, and SacII (60 units each, overnight, at 37 °C) and run in a 0.8% agarose gel. The gel was blotted to a nylon membrane (Magna, 0.45 μm; GE Osmonics Labstore) and hybridized with a ^32^P-labeled probe corresponding to *mariner* transposon positions 1385–1868 (see Materials and Methods section). The blot was dried and scanned with a Typhoon Trio phosphorimager (Amersham Biosciences). M, 1-kb plus DNA ladder (Invitrogen).
(4.5 MB TIF)

Figure S3 Immunoblots of GFP/LtrA Expressed from pACD2X-GFP/LtrA in Wild Type and Disruptants Samples were from fluorescence microscopy experiments in which wild-type HMS174(DE3) (WT) and the indicated disruptants containing pACD2X-GFP/LtrA were induced with 500 μM IPTG at 30 °C (Figures 2 and 5). Top, immunoblots of GFP/LtrA probed with anti-GFP antibody (JL-8; BD Biosciences). Bottom, parallel gels stained with Coomassie blue. Arrows to the left of the gel indicate positions of size markers (Kaleidoscope Prestained Standard; Bio-Rad). Independent repeats of the experiment gave similar results. In one experiment, the GFP/LtrA expression level in the *gppA* disruptant appeared slightly higher than that in the wild type.
(3.6 MB TIF)

Figure S4 PCR Analysis of Disruptants Containing *mariner* Transposon Insertions Using Primers Flanking the Disrupted Genes PCRs were done on genomic DNA isolated from each strain with an annealing temperature of 60 °C, using the following P1 and P2 primers specific for each gene: *gppA*, gppA1440 (5′-CGTGCCAGAGATGACATTACAGGCTAACC-3′) and gppA-1600 (5′-GATGCGTCAGCATCGCATCCGGCAC-3′); *uhpT*, uhpT1310 (5′-GCCAAGTTAGGTCTGGGAATGATTGCCG-3′) and uhpT-1560 (5′-GGCGAGAAGTTTGCCTTCACTACGCTGG-3′); *wcaK*, wcaK50 and wcaK-350 (Figure S2); *ynbC,* ynbC990 and ynbC-1670 (Figure S2); *zntR*, zntR-420 and yhdN30 (Figure S2). The PCR products were run in a 1% agarose gel, which was stained with ethidium bromide. M, 1-kb DNA ladder (Invitrogen). The prominent smaller band in the lane for the *gppA* disruptant contains primer dimers. The light band that comigrates with the wild-type band in the *zntR* disruptant (star) has a 4-bp insertion (ACAG) at the mariner transposon-insertion site (nucleotide position 171 counting from the A of the ATG initiation codon). This band presumably results from transposon excision and was found in multiple repeats with individual *zntR* disruptant colonies. Analogous bands due to transposon excision were not seen in the other disruptants.
(5.3 MB TIF)

Figure S5 DNA Target Site Sequences and Base-Pairing Interactions for Targetrons Used for Gene Disruptions Retargeted Ll.LtrB-ΔORF introns (targetrons) are designated by a number that corresponds to the nucleotide position 5′ to the insertion site in the target gene's coding sequence, followed by “a” indicating the antisense strand. DNA target sequences are shown from positions −30 to +15 from the intron-insertion site, with nucleotide residues that match those in the wild-type Ll.LtrB intron target site highlighted in gray in the top strand. The intron-insertion site (IS) in the top strand and the IEP cleavage site (CS) in the bottom strand are indicated by arrowheads. Targetron LacZ-1063a was expressed from pACD2X [51], and targetrons Ppk-1140a, Ppx-1051a, and RelA-733a were expressed from pACD-Kan^R^-RAM. The latter plasmid is a derivative of pACD2X in which the Ll.LtrB-ΔORF intron contains a modification of a previously constructed *kan^R^* retrotransposition-indicator gene [52] inserted at the MluI site in intron domain IV. Targetrons were used for E. coli gene disruption as described ([8], see also http://www.sigmaaldrich.com/Area_of_Interest/Life_Science/Functional_Genomics_and_RNAi/TargeTron.html). Prior to analysis of the disruptants, the pACD-Kan^R^-RAM donor plasmid, which carries a *cap^R^* marker on the vector backbone, was cured by transforming the strain with an incompatible Amp^R^ plasmid pACYC177, followed by growth on LB medium containing ampicillin. Targetron disruptions were confirmed by PCR and sequencing across the targetron-integration junctions and by Southern hybridization to verify a single targetron integration at the desired site (unpublished data).
(5.3 MB TIF)

Figure S6 Immunoblots of GFP/XapR Fusion Protein Expressed from pAC-GFP/XapR in Wild-Type and Disruptants Protein samples were from a fluorescence microscopy experiment in which wild-type HMS174(DE3) (WT) and disruptants were induced overnight with 500 μM IPTG at 30 °C (Figure 8). Top, immunoblot of GFP/XapR probed with anti-GFP antibody (JL-8; BD Biosciences). Bottom, parallel gel stained with Coomassie blue. Arrows to the left of the gel indicate positions of size markers (Kaleidoscope Prestained Standard; Bio-Rad). Quantitation of the immunoblot showed that the relative intensities of the GFP/XapR band in the WT, *gppA*, *ppk*, and *ppx* lanes are 1:1.05:0.61:1.07. In an independent repeat of the experiment, the relative intensities were 1:1.35:0.35:0.88.
(2.2 MB TIF)

Table S1 GFP/LtrA Localization in Wild Type and Disruptants at 37 °C (36 KB DOC)

### Accession Numbers

Accession numbers for Keio deletion strains from GenoBase (http://ecoli.naist.jp/GB6/search.jsp) are *ppk* (JW2486), *ppx* (JW2487), *uhpT* (JW3641), *wcaK* (JW2030), *ynbC* (JW1407), *zntR* (JW2354), *gppA* (JD24693), and *relA/spoT* (AQ4319).

## Abbreviations

DAPI: 4′,6-diamidino-2-phenylindole
EBS: exon-binding site
GFP: green fluorescent protein
GPPA: guanosine pentaphosphatase A
IBS: intron-binding site
IEP: intron-encoded protein
IPTG: isopropyl β-d-1-thiogalactopyranoside
LB medium: Luria–Bertani medium
LtrA: Ll.LtrB group II intron-encoded protein
*oriC,*: chromosomal replication origin
poly(P): polyphosphate
RAM: retrotransposition-activated marker
RNP: ribonucleoprotein particle
RT: reverse transcriptase
TAIL PCR: thermal-asymmetric-interlaced PCR
*ter,*: replication terminus
*Tp^R^*: trimethoprim-resistance gene
