# Supplementary figures and images for: Group II Intron Protein Localization and Insertion Sites Are Affected by Polyphosphate
Source: PLoS Biol. 2008 Jun 24;6(6):e150. doi: 10.1371/journal.pbio.0060150 (PMC2435150; doi:10.1371/journal.pbio.0060150)

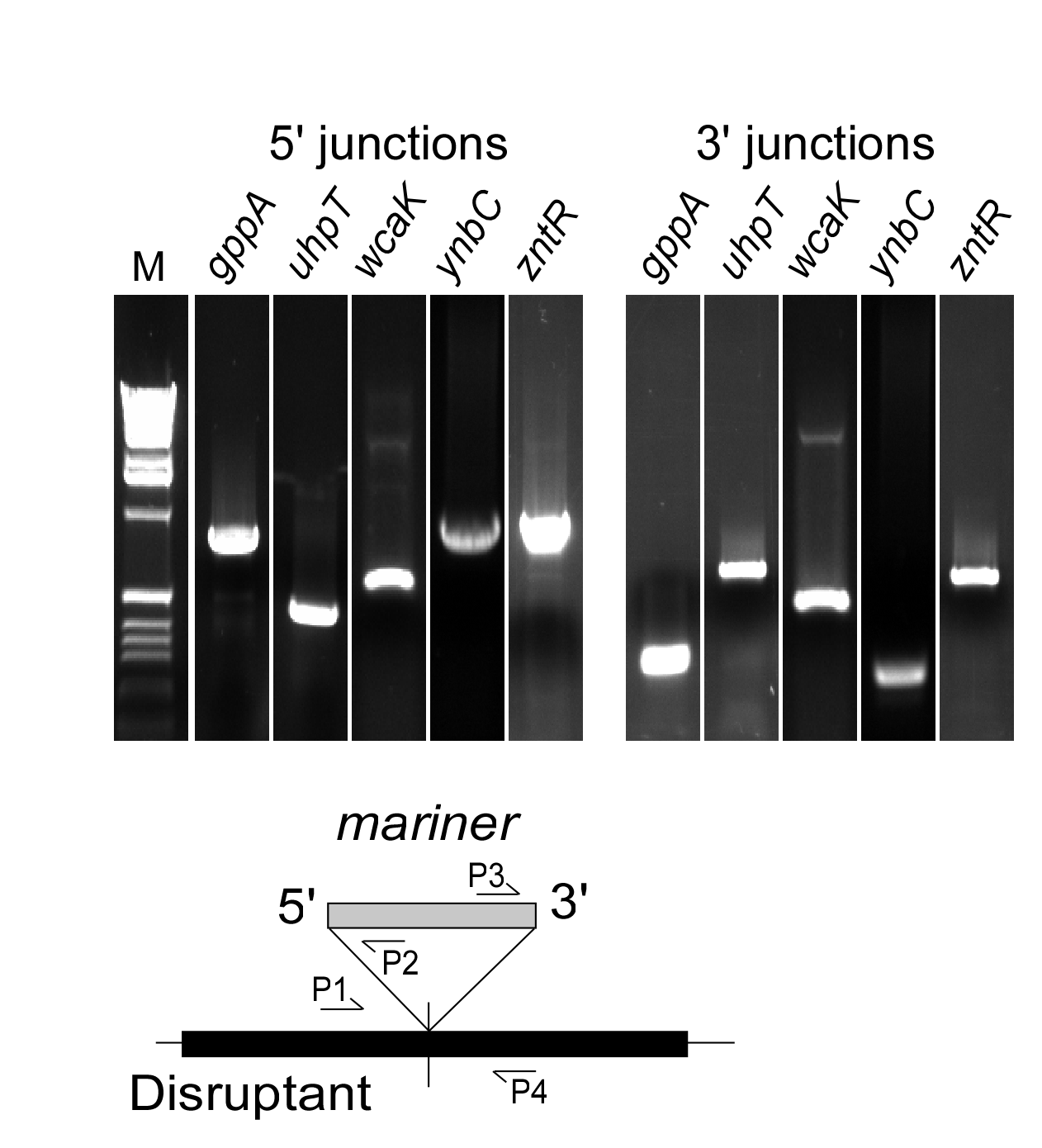

Supplement: Figure S1 — DNA isolated from the indicated disruptants by using a Genomic DNA Isolation Kit (Qiagen) was used as a template for PCR amplification of the 5′ (left) and 3′ (right) junctions of the inserted mariner transposon. The junction sequences were determined by sequencing the PCR products. The 5′ junction was amplified using primers P1 and P2, and the 3′ junction was amplified using primers P3 and P4. The gene-specific primers P1 and P4 were: gppA, gppA580 (5′-CAGTGTATGACCCTGGCGGGCGG-3′) and gppA-3end (5′-GCGTCAGCATCGCATCCGGCAC-3′); uhpT, uhpT1270 (5′-GGCTGGGCAGGCACCTTCGCCGCGC-3′) and ade1550 (5′-TGCCGTTACCCATTGCCGGGCTGATGAGC-3′); wcaK, wcaK50 (5′-GGGCAACCACACTTGCGGCAATCG-3′) and wcaK-350 (5′-CCTGATGCTGGTAGCGGCGACGGAGG-3′); ynbC, ynbC990 (5′-TTGTTCGGGACGCACTCTTCGGGCTGC-3′) and ynbC-1670 (5′-TCACGCACGAGTGAATCCATCTCCCC-3′); zntR, zntR-420 (5′-CCACTCTTAACGCCACTCGCCCCTTGTTC-3′) and yhdN30 (5′-GGCAGAGCGCCATATAGCAGAAGCGC-3′). The mariner primers P2 and P3 were: Mar-2520 (5′-GCTTCTCAGTGCGTTACATCCCTGGC-3′) and TAILP2 (5′-CGGCCGCGAAGTTCCTAT-TCCG-3′), respectively. The Figure shows PCR products run in a 1% agarose gel, which was stained with ethidium bromide. M, 1-kb DNA ladder (Invitrogen). (5.2 MB TIF) [file pbio.0060150.sg001.tif]

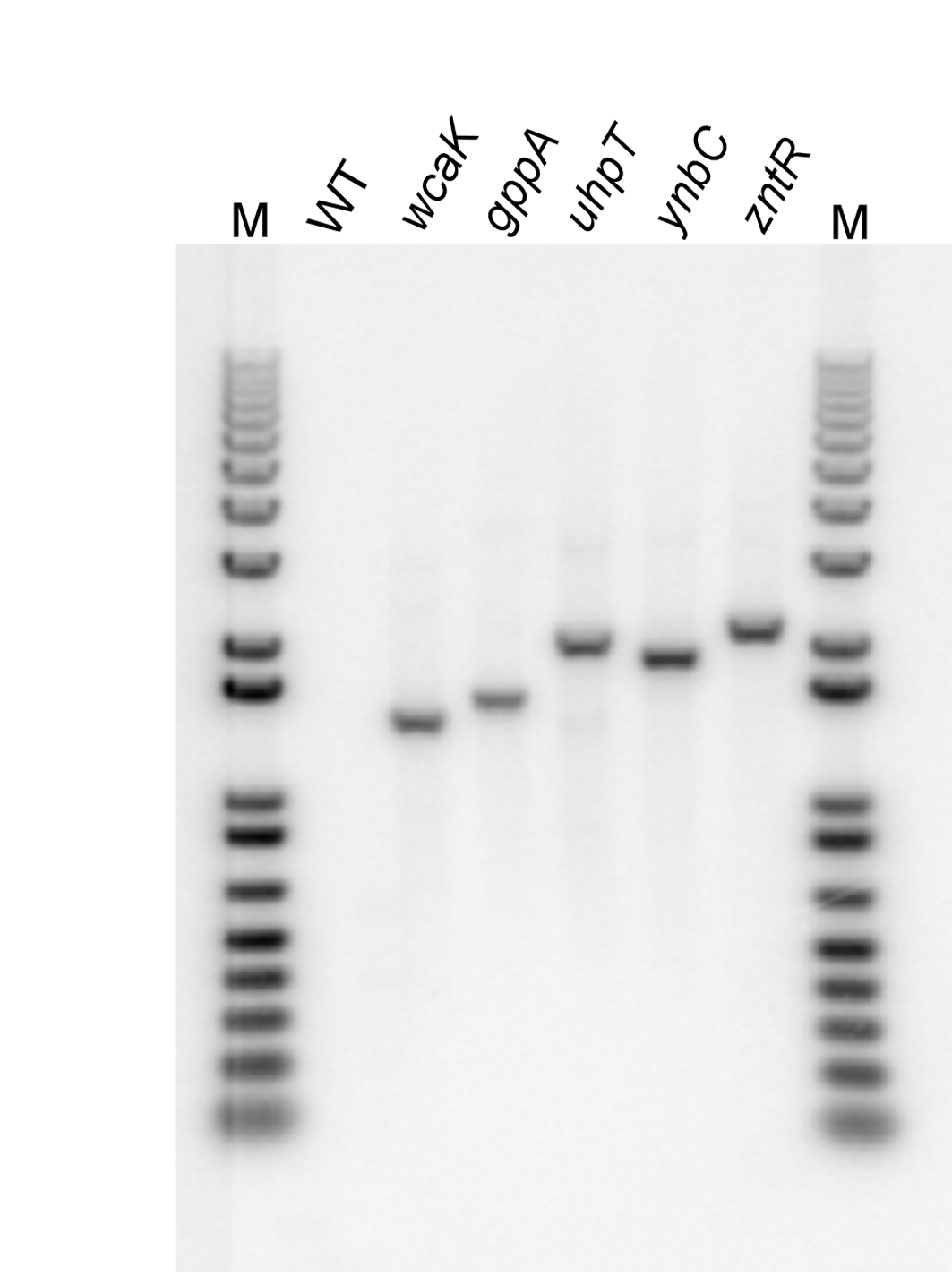

Supplement: Figure S2 — Genomic DNA (10 μg) from wild-type E. coli HMS174(DE3) (WT) and the indicated disruptants was digested with XcmI, XmaI, and SacII (60 units each, overnight, at 37 °C) and run in a 0.8% agarose gel. The gel was blotted to a nylon membrane (Magna, 0.45 μm; GE Osmonics Labstore) and hybridized with a 32P-labeled probe corresponding to mariner transposon positions 1385–1868 (see Materials and Methods section). The blot was dried and scanned with a Typhoon Trio phosphorimager (Amersham Biosciences). M, 1-kb plus DNA ladder (Invitrogen). (4.5 MB TIF) [file pbio.0060150.sg002.tif]

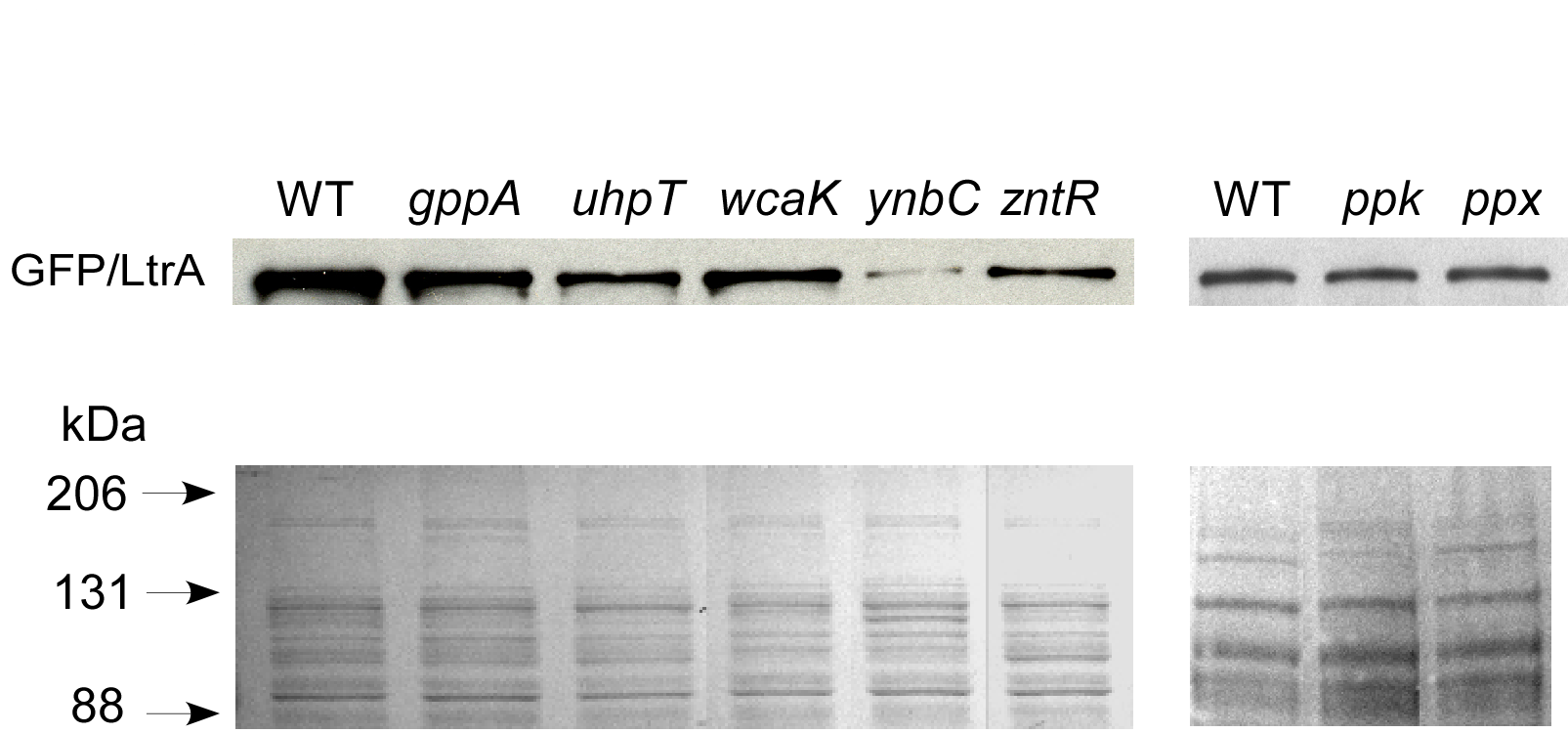

Supplement: Figure S3 — Samples were from fluorescence microscopy experiments in which wild-type HMS174(DE3) (WT) and the indicated disruptants containing pACD2X-GFP/LtrA were induced with 500 μM IPTG at 30 °C (Figures 2 and 5). Top, immunoblots of GFP/LtrA probed with anti-GFP antibody (JL-8; BD Biosciences). Bottom, parallel gels stained with Coomassie blue. Arrows to the left of the gel indicate positions of size markers (Kaleidoscope Prestained Standard; Bio-Rad). Independent repeats of the experiment gave similar results. In one experiment, the GFP/LtrA expression level in the gppA disruptant appeared slightly higher than that in the wild type. (3.6 MB TIF) [file pbio.0060150.sg003.tif]

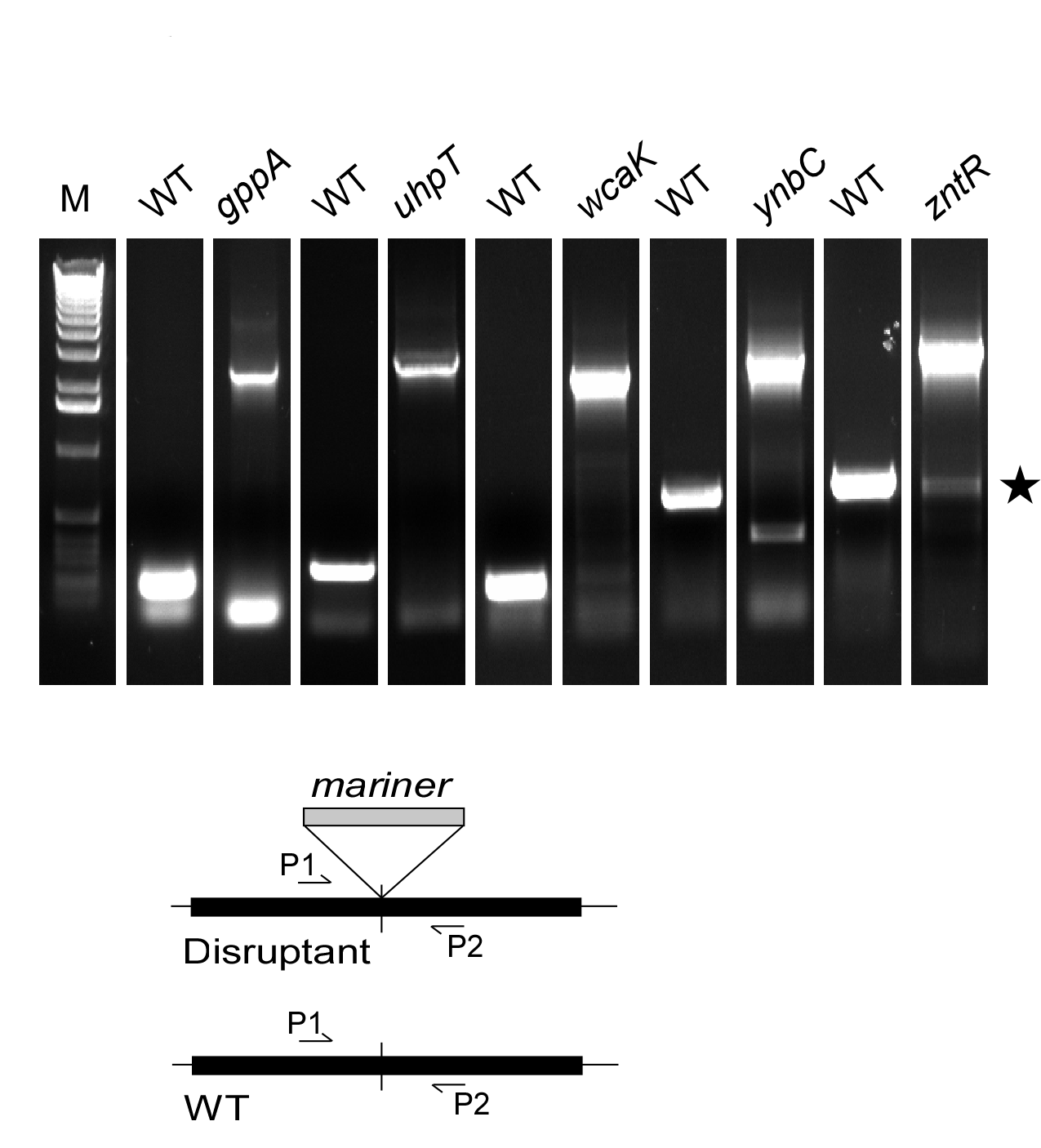

Supplement: Figure S4 — PCRs were done on genomic DNA isolated from each strain with an annealing temperature of 60 °C, using the following P1 and P2 primers specific for each gene: gppA, gppA1440 (5′-CGTGCCAGAGATGACATTACAGGCTAACC-3′) and gppA-1600 (5′-GATGCGTCAGCATCGCATCCGGCAC-3′); uhpT, uhpT1310 (5′-GCCAAGTTAGGTCTGGGAATGATTGCCG-3′) and uhpT-1560 (5′-GGCGAGAAGTTTGCCTTCACTACGCTGG-3′); wcaK, wcaK50 and wcaK-350 (Figure S2); ynbC, ynbC990 and ynbC-1670 (Figure S2); zntR, zntR-420 and yhdN30 (Figure S2). The PCR products were run in a 1% agarose gel, which was stained with ethidium bromide. M, 1-kb DNA ladder (Invitrogen). The prominent smaller band in the lane for the gppA disruptant contains primer dimers. The light band that comigrates with the wild-type band in the zntR disruptant (star) has a 4-bp insertion (ACAG) at the mariner transposon-insertion site (nucleotide position 171 counting from the A of the ATG initiation codon). This band presumably results from transposon excision and was found in multiple repeats with individual zntR disruptant colonies. Analogous bands due to transposon excision were not seen in the other disruptants. (5.3 MB TIF) [file pbio.0060150.sg004.tif]

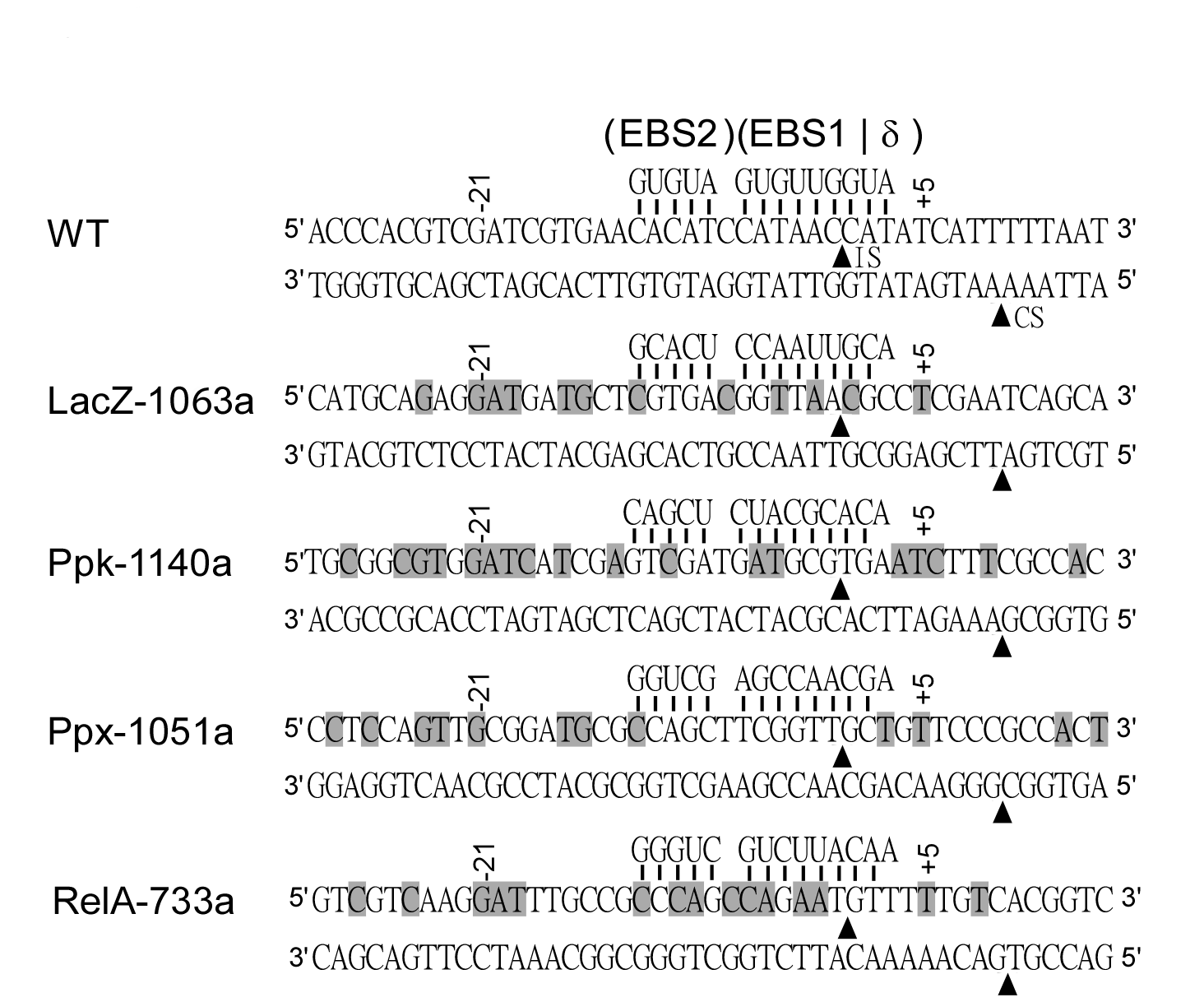

Supplement: Figure S5 — Retargeted Ll.LtrB-ΔORF introns (targetrons) are designated by a number that corresponds to the nucleotide position 5′ to the insertion site in the target gene's coding sequence, followed by “a” indicating the antisense strand. DNA target sequences are shown from positions −30 to +15 from the intron-insertion site, with nucleotide residues that match those in the wild-type Ll.LtrB intron target site highlighted in gray in the top strand. The intron-insertion site (IS) in the top strand and the IEP cleavage site (CS) in the bottom strand are indicated by arrowheads. Targetron LacZ-1063a was expressed from pACD2X [51], and targetrons Ppk-1140a, Ppx-1051a, and RelA-733a were expressed from pACD-KanR-RAM. The latter plasmid is a derivative of pACD2X in which the Ll.LtrB-ΔORF intron contains a modification of a previously constructed kanR retrotransposition-indicator gene [52] inserted at the MluI site in intron domain IV. Targetrons were used for E. coli gene disruption as described ([8], see also http://www.sigmaaldrich.com/Area_of_Interest/Life_Science/Functional_Genomics_and_RNAi/TargeTron.html). Prior to analysis of the disruptants, the pACD-KanR-RAM donor plasmid, which carries a capR marker on the vector backbone, was cured by transforming the strain with an incompatible AmpR plasmid pACYC177, followed by growth on LB medium containing ampicillin. Targetron disruptions were confirmed by PCR and sequencing across the targetron-integration junctions and by Southern hybridization to verify a single targetron integration at the desired site (unpublished data). (5.3 MB TIF) [file pbio.0060150.sg005.tif]

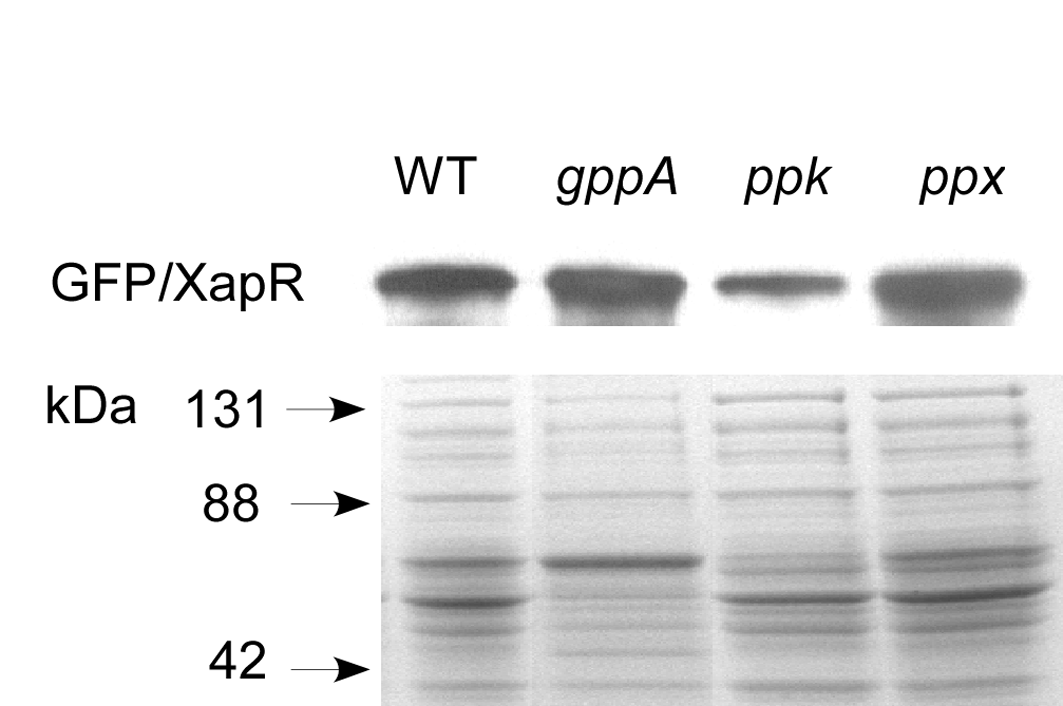

Supplement: Figure S6 — Protein samples were from a fluorescence microscopy experiment in which wild-type HMS174(DE3) (WT) and disruptants were induced overnight with 500 μM IPTG at 30 °C (Figure 8). Top, immunoblot of GFP/XapR probed with anti-GFP antibody (JL-8; BD Biosciences). Bottom, parallel gel stained with Coomassie blue. Arrows to the left of the gel indicate positions of size markers (Kaleidoscope Prestained Standard; Bio-Rad). Quantitation of the immunoblot showed that the relative intensities of the GFP/XapR band in the WT, gppA, ppk, and ppx lanes are 1:1.05:0.61:1.07. In an independent repeat of the experiment, the relative intensities were 1:1.35:0.35:0.88. (2.2 MB TIF) [file pbio.0060150.sg006.tif]
