# Supplementary material for: Group II Intron Protein Localization and Insertion Sites Are Affected by Polyphosphate
Source: PLoS Biol. 2008 Jun 24;6(6):e150. doi: 10.1371/journal.pbio.0060150 (PMC2435150; doi:10.1371/journal.pbio.0060150)
Supplement: Table S1 — (36 KB DOC) [file pbio.0060150.st001.doc]

| **Table S1.** GFP/LtrA Localization in Wild Type and Disruptants at 37^o^C | | | | |
| --- | --- | --- | --- | --- |
| **Strains** | **GFP/LtrA localization pattern (%)** | | | |
|  | **Polar** | **Diffuse (C/P)** | **Filaments** | **No Fluor.** |
| WT | 82.7 | 0.9 (0.3/0.6) | 0 | 16.4 |
| *gppA* | 40.6 | 28.1(16.8/11.3) | 11.0 | 20.3 |
| *uhpT* | 45.3 | 30.7 (9.1/21.6) | 0.5 | 23.5 |
| *wcaK* | 37.1 | 30.8 (19.8/11.0) | 15.1 | 17.0 |
| *ynbC* | 38.6 | 36.6 (9.6/27.0) | 0 | 24.8 |
| *zntR* | 46.7 | 33.4 (10.4/23.0) | 1.9 | 18.0 |

Cells containing pACD2X-GFP/LtrA were induced with 100 μM IPTG at 37^o^C overnight, and ≥ 200 cells of each strain were examined by fluorescence microscopy to characterize GFP/LtrA localization patterns. Patterns are characterized as polar, diffuse, filamentous, or no detectable fluorescence above background. For cells with diffuse GFP/LtrA localization, the percentages showing completely (C) or partially (P) diffuse fluorescence patterns are indicated in parenthesis. Filamentous cells showed either multiple foci, diffuse GFP/LtrA fluorescence, or a combination of the two.
